# Criticality Is an Emergent Property of Genetic Networks that Exhibit Evolvability

**DOI:** 10.1371/journal.pcbi.1002669

**Published:** 2012-09-06

**Authors:** Christian Torres-Sosa, Sui Huang, Maximino Aldana

**Affiliations:** 1 Instituto de Biotecnología Universidad Nacional Autónoma de México, Cuernavaca, Morelos, México; 2 Instituto de Ciencias Físicas, Universidad Nacional Autónoma de México, Cuernavaca, Morelos, México; 3 Institute for Systems Biology, Seattle, Washington, United States of America; 4 FAS Center for Systems Biology and Rockefeller Center for Latin American Studies, Harvard University, Cambridge, Massachusetts, United States of America; University of Chicago, United States of America

## Abstract

Accumulating experimental evidence suggests that the gene regulatory networks of living organisms operate in the critical phase, namely, at the transition between ordered and chaotic dynamics. Such critical dynamics of the network permits the coexistence of robustness and flexibility which are necessary to ensure homeostatic stability (of a given phenotype) while allowing for switching between multiple phenotypes (network states) as occurs in development and in response to environmental change. However, the mechanisms through which genetic networks evolve such critical behavior have remained elusive. Here we present an evolutionary model in which criticality naturally emerges from the need to balance between the two essential components of evolvability: phenotype conservation and phenotype innovation under mutations. We simulated the Darwinian evolution of random Boolean networks that mutate gene regulatory interactions and grow by gene duplication. The mutating networks were subjected to selection for networks that both (i) preserve all the already acquired phenotypes (dynamical attractor states) and (ii) generate new ones. Our results show that this interplay between extending the phenotypic landscape (innovation) while conserving the existing phenotypes (conservation) suffices to cause the evolution of all the networks in a population towards criticality. Furthermore, the networks produced by this evolutionary process exhibit structures with hubs (global regulators) similar to the observed topology of real gene regulatory networks. Thus, dynamical criticality and certain elementary topological properties of gene regulatory networks can emerge as a byproduct of the evolvability of the phenotypic landscape.

## Author Summary

Dynamically critical systems are those which operate at the border of a phase transition between two behavioral regimes often present in complex systems: order and disorder. Critical systems exhibit remarkable properties such as fast information processing, collective response to perturbations or the ability to integrate a wide range of external stimuli without saturation. Recent evidence indicates that the genetic networks of living cells are dynamically critical. This has far reaching consequences, for it is at criticality that living organisms can tolerate a wide range of external fluctuations without changing the functionality of their phenotypes. Therefore, it is necessary to know how genetic criticality emerged through evolution. Here we show that dynamical criticality naturally emerges from the delicate balance between two fundamental forces of natural selection that make organisms evolve: (i) the existing phenotypes must be resilient to random mutations, and (ii) new phenotypes must emerge for the organisms to adapt to new environmental challenges. The joint effect of these two forces, which are essential for evolvability, is sufficient in our computational models to generate populations of genetic networks operating at criticality. Thus, natural selection acting as a tinkerer of evolvable systems naturally generates critical dynamics.

## Introduction

Determining the evolutionary processes that have generated both the structural and dynamical properties observed in the gene regulatory networks of modern organisms remains a central problem in biology [1]. When analyzing the most complete data of features of gene regulatory networks available to date, two striking properties are immediately apparent. First, on the structural side, these networks exhibit hub-like structures characterized by the presence of few global regulators, namely, a few genes that regulate the expression of a large fraction of other genes in the network [2]. For instance, in the *Escherichia coli* gene transcription network, seven global regulators regulate the expression of more than 60% of the genes in the entire network [3]. Second, on the dynamical side, recent analyses of patterns of transcriptome changes in several organisms reveal that gene regulatory networks operate in a critical regime, i.e. close to a phase transition between ordered and chaotic dynamics [4]–[9]. However, how genetic networks with hub-like structures and critical dynamics emerged in evolution remains elusive. It is not known whether these two “emergent” properties, one structural and the other dynamical, are related to each other or if they were directly selected for and whether they are the result of completely independent selection processes or constraints.

### Two trade-offs related to stability and change

Several models of network growth and evolution have been devised to generate networks with specific topological properties (such as hub-like structures, [10], [11]) or with a particular type of dynamical behavior [12], [13]. The ‘dynamics’ of a genetic network, that is, the collective change of gene expression of all the genes in the network, (i.e. of the gene expression pattern), is obviously the more appropriate phenotype on which evolution acts than the topology itself. However, networks are often, again, trained explicitly to exhibit a particular behavior, such as robust dynamics under certain kinds of perturbations [14]–[18], or to perform some arbitrarily imposed task [19]. Usually, the training is achieved by selecting the networks that score highest with respect to a suitable fitness function. In contrast to such explicit targeting of particular phenotypes as endpoints we propose that an elementary and more encompassing set of constraints must be taken into account, which is epitomized in these two distinct trade-offs of opposing features:

- From the perspective of ontogenesis one is interested in properties that ensure *phenotypic robustness* and at the same time *flexibility* given the conditional need of a network to produce multiple phenotypes (stable gene expression patterns). *Robustness* is the resilience of a given gene expression pattern to environmental perturbations of gene expression. *Flexibility* by contrast refers to distinct changes of gene expression patterns (phenotype switching) during development and to cope with environmental fluctuations. We will refer to this balance between phenotypic robustness and flexibility as the *developmental trade-off*.
- From the evolutionary perspective, *mutational robustness* (resilience of the phenotype to alterations of the genome) is essential in order to maintain vital traits, but at the same time, mutations must also be able to generate *new phenotypes (phenotypic innovation)*. We will refer to this second balance, consisting in the coexistence of mutational robustness and phenotypic innovation, as the *evolutionary trade-off*.

The second trade-off epitomizes the two central properties that underlie evolvability [20]–[22]. Concretely, the evolutionary trade-off, the central subject of this study, implies that when new phenotypic traits are developed, the old, useful traits do not disappear but are conserved or transformed into something similar. A fundamental question in evolutionary biology is whether the evolutionary trade-off is the result of adaptation by natural selection, or arises through non-adaptive mechanisms. There is a great amount of evidence suggesting that evolvability itself is a selectable trait and hence, evolvability evolves [23]–[26]. However, the mechanisms through which evolvability evolves are still under debate.

The two dualisms, the evolutionary trade-off and the developmental trade-off, are of course interconnected in the sense that the latter is an adaptive phenotype of the evolving individual, that is, it is shaped by selection pressure. Indeed it was precisely because of the developmental trade-off that critical dynamics has been hypothesized to play an important role in evolution [27]–[29].

### Critical dynamics

Critical dynamical systems operate at, or close to, a phase transition between ordered and chaotic dynamics. They exhibit a series of very remarkable properties that would be difficult to explain in the absence of criticality, such as collective response to external stimuli without saturation [30]–[32], optimal computational capabilities [33], fast information storage, transfer and processing [34], etc. In fact, the existence of critical dynamics in living systems has been increasingly recognized as an important property that confers collective behavior over many different scales [35]. In general terms, critical dynamics in gene regulatory networks implies that perturbations of gene expression would neither amplify and percolate through the system (manifest by the overwhelming divergence of the trajectories of any two initial states, as seen chaotic systems) nor would they immediately “die out” (manifest by the overwhelming convergence of the trajectories of any two initial states, as seen in ordered systems). In computational models gene regulatory networks that operate in the dynamically critical regime (between order and chaos) have been shown to exhibit both homeostasis (robustness of gene expression states) and developmental progression (change of gene expression state), thus achieving some sort of optimization (or balance) in the developmental trade-off [4], [5], [27]–[29]. Thus, criticality is a mechanism that, within an organism, engenders dynamical robustness to the network while at the same time allowing the network to respond to developmental perturbations.

Therefore, for the development of the individual organism there are compelling reasons to assume that dynamical criticality in their genetic networks is a desirable property. This may explain why experimentally observed gene expression patterns in several organisms indicate that the regulatory networks indeed operate in the critical regime [4]–[9]. However, to our knowledge in previous work on dynamical criticality in genetic networks, this property has either been taken for granted or externally imposed by adjusting the value of a network control parameter that is known to operate the order-chaos phase transition. In these studies the networks are constructed by design to be in the critical phase, (or in the ordered or the chaotic phase) followed by the analysis of their properties and contribution to evolution [30]–[34]. In a case where criticality in fact emerged was due to imposed explicit “rewiring” rules [36]. However, little is known about how dynamical criticality emerges without such explicit enforcement but in an evolutionary process that is inescapably subjected to the constraints of evolvability.

Therefore, here we ask: what is the role of evolution in poising gene regulatory networks at the critical phase? How does a gene regulatory network evolve a structure that confers criticality in the first place? What properties must be selected for in order for a non-critical network to become critical?

In this work we evolve populations of simulated gene regulatory networks and show that criticality is profoundly linked to evolvability. More specifically, we show that critical dynamics, and hence the developmental trade-off in genetic networks, naturally emerge as a robust byproduct of the evolutionary processes that select for evolvability and optimize the evolutionary trade-off. Furthermore, the emergence of criticality occurs without fine-tuning of parameters or imposing explicit selection criteria regarding specific network properties.

### Boolean networks as models of gene regulation

As a model for gene regulatory networks we use the Boolean network model proposed by Kauffman [27]–[29], [37]–[40]. It has been firmly demonstrated that this model of complex networks effectively captures essential aspects of gene regulation at the promoter which involve highly cooperative, non-linear, conditional relationships. These mechanisms are adequately encoded by logical functions that can reproduce well the observed dynamics of real networks with partially known topology [29], [39], [40]. But more important, the mapping between network architecture space and dynamical regimes is well known for Boolean networks, such that a randomly generated ensemble of networks can be controllably constrained by network architecture parameters. In brief, a Boolean network is defined by a set of nodes, 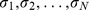, representing the genes, each acquiring the values 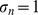 and 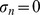, corresponding to the two states of gene expression: either the gene is expressed (gene locus is active) or it is not expressed. The value of each node 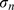 is determined by a set of 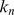 other nodes in the network, the regulators of 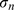, denoted as 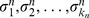. The network dynamics are then given by the simultaneous update of all the network elements according to the equation
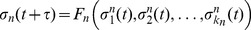
(1)where 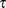 is an average response time (usually taken as 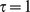) and 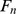 is a Boolean function constructed according to the activating or inhibitory nature of the regulators of 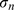. For specific networks of real organisms, the connections and Boolean functions can be chosen to capture the molecular biology of the regulatory mechanism that is often known in the form of a qualitative proposition that contain logical relationships. Such modeling approach has been shown to reproduce the observed gene expression patterns in a variety of organisms. Since we are not interested in a particular network of a specific organism, in the initial population we use random networks in which the 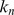 upstream regulators of a given gene 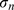 are chosen randomly. The Boolean functions of each gene 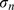 are also assigned randomly in a way such that for each of the 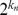 activity configurations of the 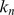 regulators, the Boolean function outputs to 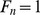 with probability *p* and to 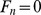 with probability 1-*p*. The value of *p*, referred to as the ‘bias’ of the Boolean function, is one of the key parameters of the global gene network architecture that influence the dynamics.

Since the discrete valued network with *N* genes has a finite number 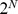 of possible dynamical states which form the state space, and since the dynamics given by Eq. (1) is deterministic, any trajectory will eventually visit a state previously visited and enter into a periodic pattern of expression that repeats over and over again. More than one periodic pattern can exist for the same network. Such periodic patterns are the dynamical *attractors* of the network which thus consist of either a set of states that form a “state cycle” (analogous to limit cycle in continuous systems) or of a single steady state (analogous to a fixed-point attractor). The set of states that converge to the same attractor constitute its *basin of attraction*.

Three important aspects of Boolean networks are relevant here. First, the dynamical attractors of the network correspond to the distinct functional phenotypic states of the cell, such as cell types, as has now been experimentally demonstrated [39]–[42]. Therefore, the set of all the dynamical attractors of a given network constitutes its *phenotypic landscape* (in the sense of Waddington's epigenetic landscape [43]) which we refer here as the *attractor landscape*. Second, two broad regimes of dynamical phases that depend on global network topological parameters can be identified: the ordered and the chaotic phase [7], [27]–[29], [37], [38]. Networks in the ordered phase are dynamically too rigid because in such networks any perturbation in the initial condition eventually disappears and the networks relax back to the pre-perturbation state. In the extreme case, all transients converge to only one attractor state, thus permitting only one stable phenotype. By contrast, networks in the chaotic phase typically have large numbers of attractors and/or vastly long transients and are extremely sensitive to small perturbations, making all network states very unstable. And third, there is a continuous phase transition between the ordered and chaotic phases that is characterized by a nontrivial critical point. Networks that operate close to this critical point display a series of interesting properties of complex systems, such as the maximization of information processing needed for ontogenetic complexity [34], [44].

One order parameter that determines in which dynamical phase the network operates is the *average network sensitivity S* defined as [45]

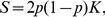
(2)where *K* is the average number of upstream regulators per gene and *p* is the fraction of positive (activating) regulations in the set of all Boolean functions in the network. If 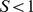 the network is in the ordered phase, and if 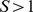 it is in the chaotic phase [7], [27]–[29], [37], [38]. The critical phase is attained at 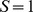.

Note that the above definition of the ordered, critical and chaotic phases refers to the level of dynamics, namely, to the response of the network to transient perturbations. However, we recently found that classification of networks into these dynamical regimes has an interpretation that extends to the evolutionary time scale. Specifically, the probability for a change of the attractor landscape, thus of the global phenotypic behavior, following simple mutations to the network structure is very low for networks in the ordered phase and is very high for chaotic networks [46]. Hence, networks in the ordered regime are not evolvable because they absorb the effect of mutations in a large space of neutral mutations. On the other hand, those in the chaotic regime are highly innovative in the sense that their attractor landscape undergoes large scale changes following the small mutations—but they lack mutational robustness. Critical networks are peculiar because following a simple mutation, new attractors emerge with high probability while conserving existing attractors. Thus, critical networks are both robust and capable of useful innovation, hence are evolvable. In view of this relationship between criticality and evolvability, the question of how genetic networks became dynamically critical is thus linked to the question of how evolvability arose through evolution. Could the evolution of evolvability account for the evolution of criticality?

## Results

### Evolutionary algorithm

**Figure 1:**
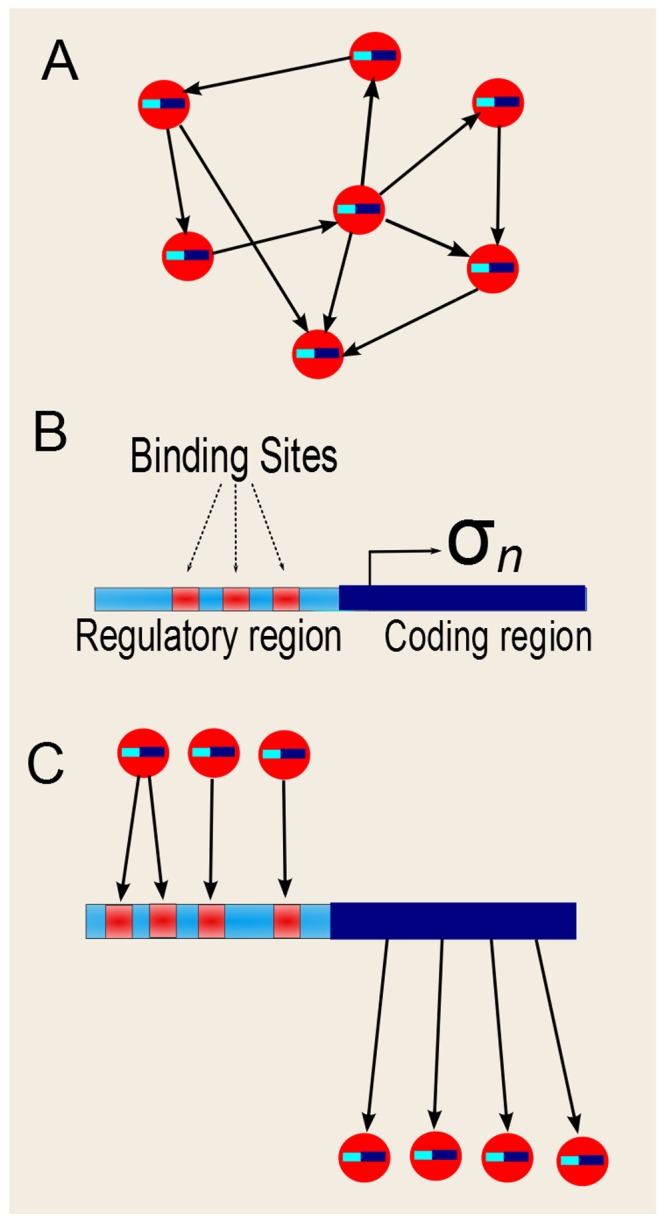
Gene structure. (A) Schematic representation of the network, showing that each node (circles) “contains” one gene (little bars inside the circles). The arrows represent the regulatory interactions between the genes. (B) Each gene 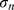 is composed of a regulatory region and a coding region. The regulatory region contains binding sites which can be added to, or removed from, the regulatory region with the same probability. (C) The binding sites in the regulatory region determine the regulators of 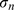 (its input connections). There can be more than one binding site per regulator (as the regulator on the very left), although at the beginning of the evolutionary process the initial networks have only one binding site per regulator. The regulatory region of 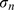 determines which other genes it regulates.

We simulated the evolution of genetic networks in a starting population of *M_0_* = 1000 different random Boolean networks each with *N* = 10 nodes. Initially, all nodes have exactly 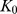 upstream regulators and the Boolean functions have a bias *p* = 0.5. Hence, the sensitivity of the networks in the first generation is entirely determined by the initial network connectivity 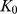 through 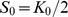 (Eq. 2). We mutate the networks in the population by implementing a mutation algorithm that captures fundamental properties of biological genome growth. Specifically, each node represents a gene that is composed of a regulatory region and a coding region, as illustrated in Fig. 1, and mutations can occur in any of these two parts. Mutations in the regulatory region consist in the addition or deletion of binding sites to DNA. These mutations change the way in which the node is regulated by its upstream regulators (see Fig. 1B and the Methods section for a detailed description of the mutation algorithm). Briefly, mutations in the regulatory region of a given node 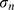 can eliminate or establish regulatory inputs from existing or new upstream regulators, respectively, or produce changes in its Boolean function. On the other hand, mutations in the coding region of node 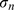 change the spectrum of its target nodes, which translates into the gain of new targets, loss of existing ones or changes in the Boolean functions of the targets of 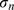. Finally, network growth is implemented by simulating the evolutionary mechanism of gene duplication followed by divergence [47]. This is done by randomly choosing one node in the network and duplicating it, along with its network connections, thus increasing the number of nodes in the network from *N* to *N*+1. We then simulate gene divergence by mutating either the regulatory or the coding regions of the duplicated node 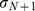. Due to computational limitations, networks were allowed to grow up to a maximum size of *N* = 100. It is important to mention that even if the mutation algorithm effectively implements the random addition or removal of input or output connections in the network or changes in the Boolean functions of the nodes, the probabilities for these effective mutations to occur change from one node to another and also in time. The reason for this is that these effective probabilities depend on the network size and on the number of binding sites that each node has. Therefore, in the Materials and Methods section we present the mutation algorithm in terms of the probabilities for adding and removing binding sites to the regulatory regions of the nodes because these probabilities remain constant throughout time and across the network elements.

### Darwinian selection

Mutations in the regulatory or coding regions of the nodes occur randomly with a mutation rate 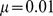 per gene per network per generation. Once a given gene is selected to be mutated, one of the mutations [(1)–(6), as described in the Methods section] is performed. Let 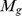 be the number of networks in the population at generation 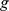. Then, on average 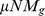 networks undergo mutations in one of their genes and are subjected to selection. To select for mutational robustness we evaluate at each generation whether or not the mutated networks conserve the attractors that they had before the mutation and eliminate from the population those networks which do not conserve all their attractors. By attractor conservation we mean strict maintenance of identity of attractor states. If after the mutations one of the network attractors changes even only by one bit in its binary states, that change is enough to declare that attractor as non-conserved. Only the networks that conserve all the attractors they had before the mutations will pass to the next generation. We will refer to this selection process as the *attractor conservation criterion* (ACC). The elimination of the networks that do not satisfy this criterion reduces the population size to a new value 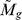. If 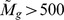 the population is still big enough and we just go to the next generation without replicating any network. However, if 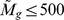 we replicate the surviving networks in order to restore the population to its original size *M_0_* = 1000 (or to a size as close as possible to 1000). For this we have to decide whether all the networks will equally reproduce, or if some networks will reproduce more than others. In the latter case, we have to define a fitness function which will determine the number of copies (daughters) generated by each of the surviving networks to maintain the population size. Let us assume that we already have a fitness function that assigns a fitness value 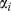 to the *i* ^th^ surviving network in the population, with larger values of 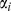 corresponding to fitter networks. In the next section we give a precise definition of 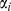 based on the gene expression variability within the attractors but for the time being let us just assume that 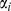 is already given. Then, if 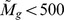 the *i* ^th^ surviving network will produce 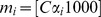 daughters, where the function 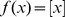 gives the closest integer to *x* and the normalization constant 
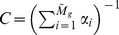
 guarantees that the new population size 
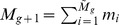
 is as close to 1000 as possible. (We cannot always make it exactly equal to 1000; for instance, if 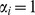 for all networks and 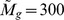, then triplicating each surviving network will restore the population to 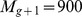). We will refer to this replication mechanism as the *α*-fitness criterion.

In order to simulate phenotypic innovation, every 2000 generations all networks in the population simultaneously undergo a gene duplication-divergence event. Therefore, the duplication rate is in the order of 10^−5^ per gene per generation which is in the broad range of estimates based on genome sequence data and similar to numbers used in previous models of network evolution [48]–[50]. After this event the only networks that survive and pass to the next generation are the ones that, *in addition* to fulfilling the ACC, also generate new attractors. We will refer to this selection rule as the *attractor innovation criterion* (AIC). Therefore, under this criterion we eliminate from the population all the networks which, after the duplication event, either do not satisfy the ACC or do not generate new attractors (even if some of these latter networks fulfill the ACC). In principle, any mutation can generate new attractors. However, we evaluate the emergence of new attractors only after gene duplication events because it is known that the average number of attractors increases with the size of the network *N* [51], [52]. Therefore, it is much more likely to find new attractors when the network grows. It is worth noting that before the duplication event the network had *N* nodes, and after the duplication it has *N*+1. Hence, to compare the attractors of the network before and after the duplication event in order to check for conservation or innovation, we only take into account the first *N* nodes of the network (genome) which are the ones common before and after the duplication event, and ignore the value of the (*N*+1)^th^ node which represents the new gene resulting from the duplication event. Another important point to mention is that, due to computational limitations, in our simulations the whole set of attractors in the attractor landscape was determined only for small networks (*N*<25). For large networks (*N*≥25) a thorough search of the state space to find all the attractors is very time consuming and not feasible. Therefore, to assess attractor innovation in large networks we sampled just a subset of the 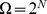 possible states. Clearly, the AIC was applied only to the attractors that were found with this subsampling. In the Methods section we explain the details of the algorithm to find new attractors.

### Fitness function based on gene expression variability

Another aspect we took into account when new attractors emerge is that the nodes in these attractors must contribute to a phenotype. In other words, as the network grows and mutates, the new nodes added to the network cannot be all frozen in state 1 or all frozen in state 0. In the attractors some of the new nodes must be 1 and some others must be 0 (or they can oscillate as well). Without this condition, the growing part of the network would carry no useful information. Networks whose attractors have no information are biologically irrelevant, as it is known that real organisms have gene expression profiles with high information content [6]–[8]. Thus, the information content of the attractor states can be used to define the aforementioned fitness function that determines the replication rate of the networks. In order to do so, we define the average gene expression variability of the network attractors as 
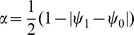
, where 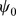 and 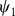 are the average fractions of 0's and 1's in all the states of all the attractors of the network (clearly, 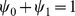). Thus, 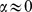 if almost all the nodes in the attractors are in only one state (either 0 or 1), whereas 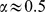 if more or less half of the nodes in the attractors are in the state 1 and the other half in the state 0. In order to implement this phenotypic fitness after the duplication event, when restoring the population to a size close to 1000, we replicate each surviving network by a quantity proportional to its average gene expression variability 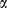. This is the *α*-*fitness* parameter that we mentioned in the previous section.

**Figure 2:**
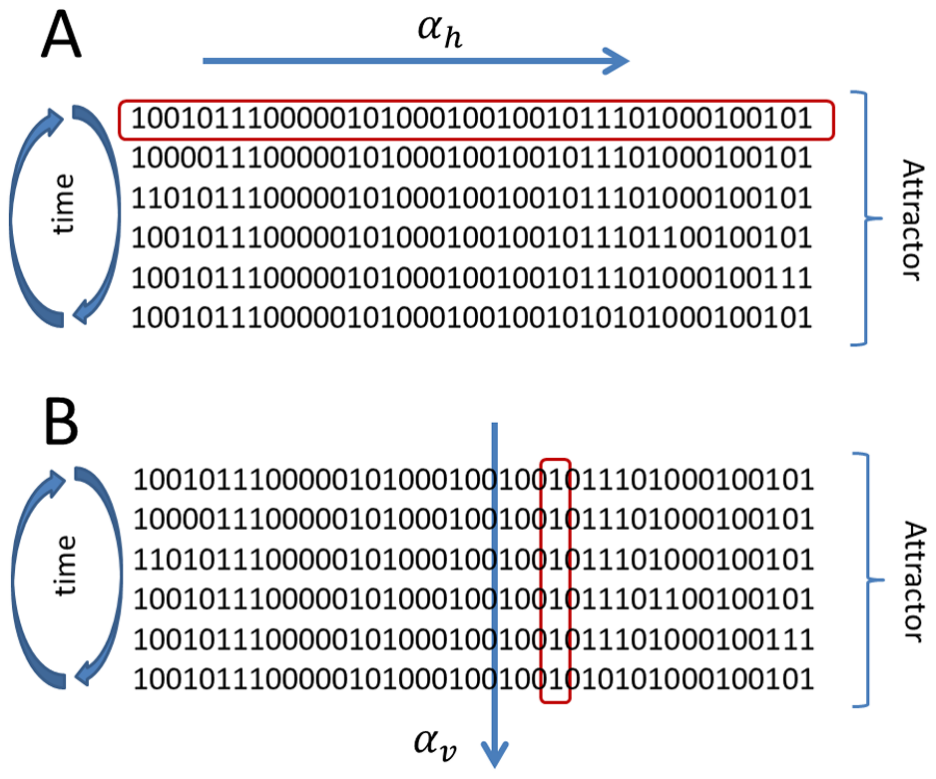
Gene expression variability *α*. This parameter, which measures whether the genes are frozen in one state, either 0 or 1, or if they more or less switch back and forth between these two states, can be computed in two distinct ways. The first way (horizontal variability 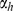) is to measure 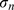 along each attractor state, as shown in A, and then average over all the attractor states and over all the attractors in the attractor landscape. The second way (variability 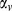) shown in B, is to measure the variability for each gene throughout time along the attractor cycle, then average over all the genes in the network and over all the attractors. This is illustrated by the particular example for the same attractor where 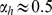, whereas 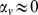.

It is important to note that there are two ways to measure the variability *α*, as illustrated in Fig. 2. The first way is to measure the variability along the *N* nodes of each attractor state (Fig. 2A), and then average over all the states in the attractor and over all the attractors in the network. We will refer to this parameter as the horizontal gene expression variability and denote it as 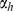. The second way is to measure the variability of each node 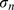 individually along the attractor cycle (Fig. 2B) and then average over all the nodes in the network and over all the attractors. We will call this quantity the vertical gene expression variability and denote it as 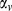. These two parameters need not give the same results, as illustrated in Fig. 2, where 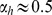 whereas for the same attractor 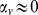. In all the numerical simulations presented here the *α*-fitness criterion was implemented using the horizontal variability 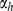.

It is also important to stress the fact that the ACC (corresponding to mutational robustness), the AIC (corresponding to phenotype innovation), and the 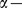 *fitness* act on the attractor landscape, which is entirely an intrinsic dynamical property of the network. Therefore, our selection criteria do not train the network to perform an arbitrary task imposed externally. On the contrary, the ACC, the AIC and the 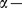 *fitness* acting together throughout the evolutionary process optimize the networks in the population with respect to the evolutionary trade-off by taking into account conservation and expansion of the network's intrinsic attractor landscape, whatever it is.

### Emergence of criticality

**Figure 3:**
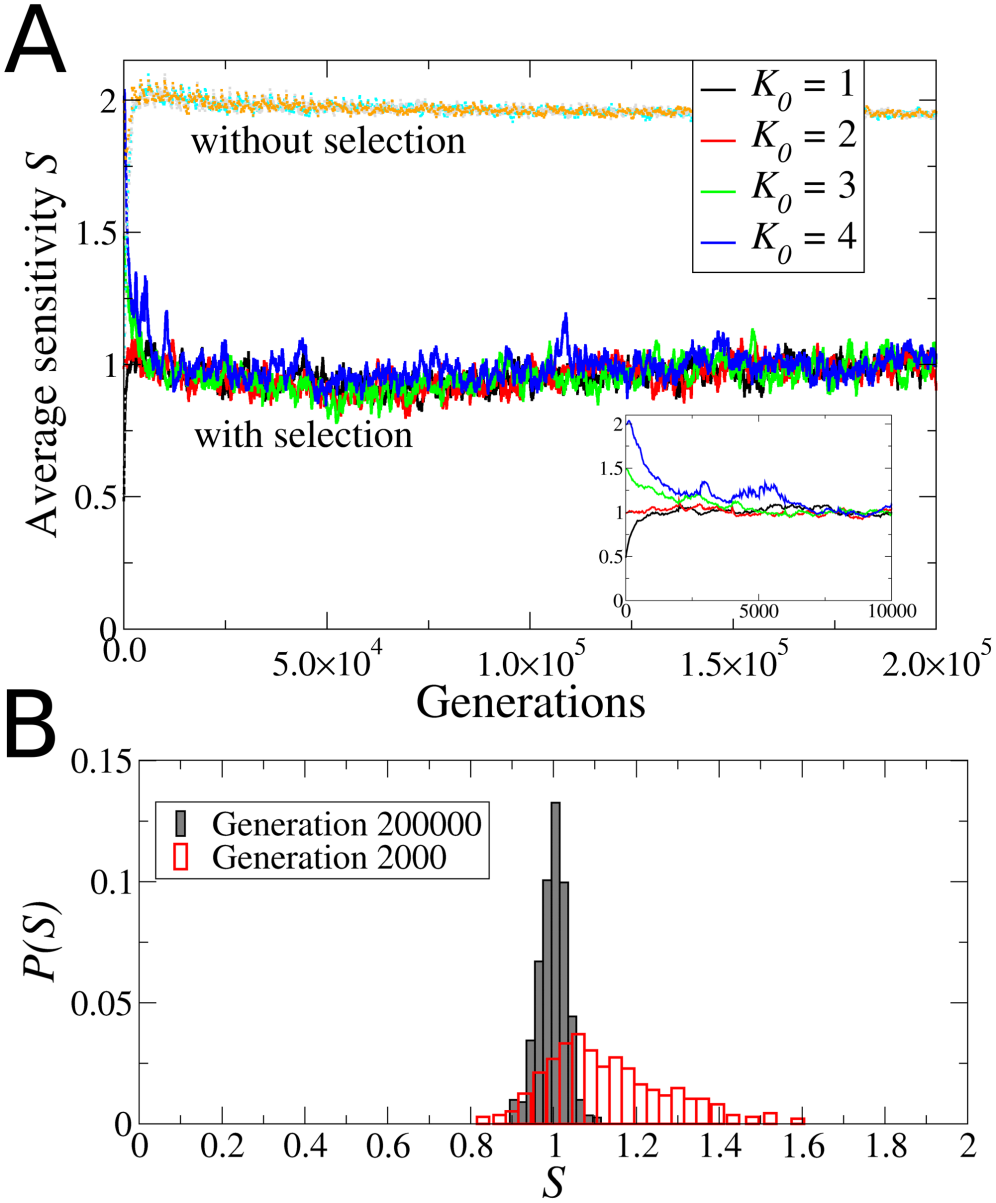
Evolution towards criticality. (A) Evolution of the average network sensitivity for four different populations, each initially composed of networks in one of the three dynamical regimes: ordered (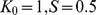, black), critical (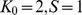, red), and chaotic (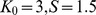, green; and 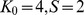, blue). Under the Darwinian selection given by the ACC and AIC, all the populations quickly become critical (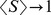), regardless of their initial dynamical regime. The inset shows that convergence towards criticality occurs during the first 10000 generation steps. The control curves (in light gray) were obtained by evolving populations without selection, and show that the mutagenic method alone drives the networks into the chaotic regime (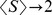). (B) Distribution of sensitivities at two different generations for the population that started with 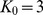 chaotic networks. In early generations 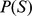 is quite broad (dashed line), reflecting a great diversity of networks. However, through evolution all the surviving networks approach criticality and the distribution 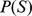 narrows down (solid line). The distribution shown here at generation 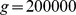 has 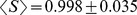.

Fig. 3A shows the evolution of the average network sensitivity 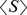 of the population, where the average is taken over all the 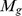 networks in the population at generation 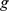. The four different curves correspond to four different starting populations, each consisting either of only ordered networks, only critical networks, or only chaotic networks, according to the initial sensitivity 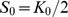. The curves that converge to 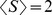 (representing chaotic dynamics) show the effect of a control algorithm in which mutations where applied without selection (all networks survive in each generation). Thus, the mutation algorithm alone does not account for the emergence of criticality because it produces chaotic networks. By contrast, when selection is present, the sensitivity of the networks in all populations converge, on average, to the value 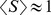, indicating evolution to criticality. Therefore, Darwinian selection, realized by the selection filters ACC and AIC, promotes the evolution of networks towards criticality. Fig. 3B shows the distribution of sensitivities 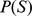 in one of the populations that started with chaotic networks (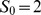) at two distinct generation times in the simulation, early (generation 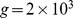) and at the end (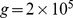). The distribution 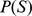 reveals that not only does the average 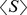 evolves towards criticality (mean 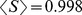) but that the initially broad diversity decreases throughout evolution (the standard deviation decreases almost one order of magnitude, from 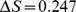 at generation 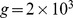 to 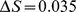 at generation 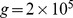). The results reported in Fig. 3A are highly reproducible. (In Fig. S1 we present similar plots for 30 more realizations of the evolutionary processes, including seven realizations for which the networks in the initial population had nodes with varying input connectivity. Additionally, in Fig. S2 we present the Derrida maps of the networks that result from the evolutionary process, which show in a more formal way that all the networks become critical. See Text S1 for a definition of the Derrida map.)

**Figure 4:**
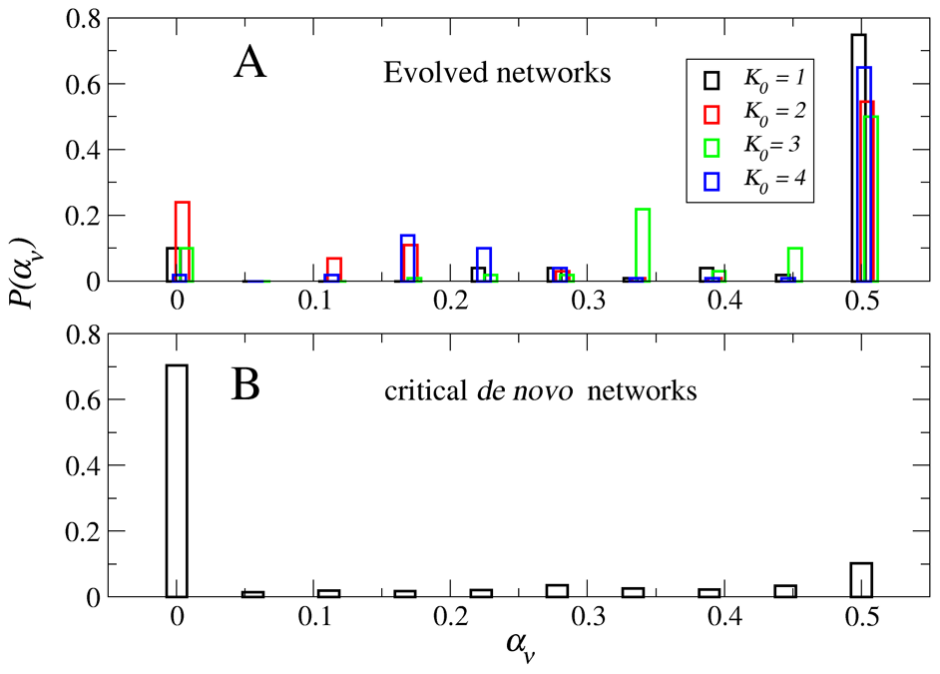
Genetic variability in the attractors. (A) Histogram 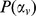 of the vertical genetic variability 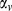 in the attractors of the evolved networks. Note that most of the genetic variability is concentrated around 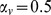, which indicates that most of the genes in the attractors of the evolved networks switch back and forth between 0 and 1 throughout time. There are almost no frozen genes in these attractors (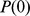 is relatively small). (B) Histogram of the vertical variability in the attractors of *de novo* critical networks that were constructed to be critical by design and did not go through the evolutionary process. Note that in this case most of the genes are frozen in time, as the largest peak at 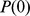 indicates.

Another important property to look at is the gene expression variability of the evolved networks. Since in our numerical simulations we used the horizontal variability 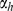 as the fitness parameter that determines the replication rate of the surviving networks, in the final population all the networks have 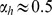, as expected (data not shown). However, it turns out that the vertical variability 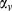 is also distributed mostly around 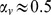, as Fig. 4A shows. This is a non-trivial result first, because there is no reason *a priori* to expect 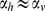, as these two quantities need not bear any relationship (see Fig. 2). But second, and more importantly, because control networks that are explicitly constructed to be critical *de novo* have a distribution of vertical variability 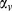 dominated by 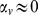, as shown in Fig. 4B. Thus, the fact that the evolved networks have both 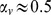 and 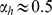, cannot be trivially explained as an inherent feature of criticality nor by selection for *α*-fitness alone. Rather, it is a result of the entire evolutionary processes.

**Figure 5:**
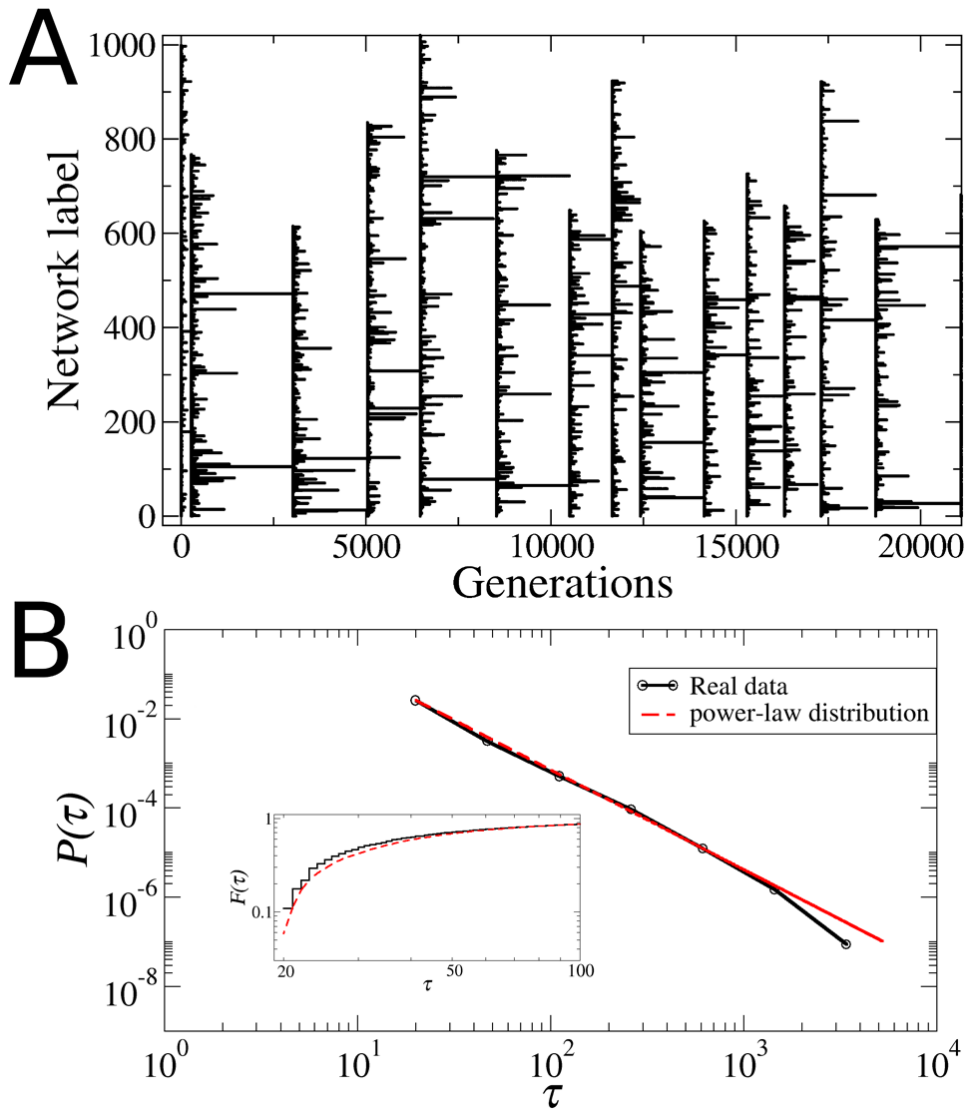
Survival times of the different strains in the population. (**A**) Plot of the network labels (strains) that are present in the population at a given generation. Each horizontal line indicates the survival time of a particular strain. The vertical lines indicate the fixation events in which all the networks in the population are relabeled after only one strain was left in the entire population. (B) Distribution 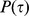 of survival times computed during 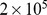 generations (black curve). This distribution was computed using logarithmic bins. Only data for 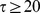 are presented because we checked the existence of strains every 20 generations. The red dashed line is the best fit which corresponds to the power-law 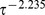. The inset shows the corresponding cumulative distribution 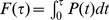, which better reveals the goodness of the power-law fit. The fact that 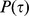 has a more or less power-law behavior implies that almost all the strains disappear from the population very quickly, whereas only very few networks are able to survive the Darwinian selection mechanism given by the ACC, the AIC and the *α*-fitness criterion.

To determine how restrictive the selection criteria, ACC and AIC, that must be satisfied for a network to survive selection, are, we measured the survival times of the networks by tracking individual networks (Fig. 5). We tracked all initial networks in the population by labeling them individually with an integer ranging from 1 to 1000 at generation 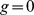. When one network is replicated into several copies the “daughter” networks acquire the same label from the “mother”. Since the networks that fail the selection criteria are removed from the population, some labels can disappear altogether from the population. This would correspond to the extinction of one lineage. If at generation *g* only one label is left in the entire population of 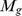 networks this can be considered the “fixation” of a particular strain in the population and we re-label the networks again from 1 to 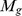. Fig. 5A shows the evolution of strains (labels) through 20,000 generations. Presence of individual strains in the population is indicated by the horizontal lines, with the longest surviving strains defining the fixation events indicated by the vertical lines. The vast majority of strains disappeared from the population very quickly while only very few strains survived for long periods. Interestingly, a goodness-of-fit test indicates that the distribution 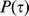 of survival times 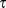 is highly consistent with a power-law, 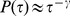 with exponent 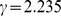 (Fig. 5B), as observed for geological life spans of genera from fossil records [53]. Whether or not 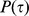 is in fact best fitted by a power-law is here not of fundamental relevance. Of significance however is the broad tail exhibited by this distribution, for it shows that the vast majority of strains disappear very quickly from the population and only very few strains are able to survive. Therefore, the results reported in Fig. 5 demonstrate that evolution towards criticality via the fitness criteria of attractor conservation and innovation, and of gene expression variability, indeed confronts the population to a series of highly restrictive selective filters (bottlenecks) through which only very few networks are able to go.

The data reported in Figs. 3 and 5 also show that, even though there is a great genotypic and phenotypic diversity in the initial population (because initially all the networks are structurally different and have different attractor landscapes), throughout generations the population passes through a series of selective filters which decrease this diversity by eliminating from the population the majority of strains. Indeed, it is clear from Fig. 5 that several fixation events occur throughout the evolutionary processes. Therefore, at the end of the simulation all networks in the population come from one common ancestor. This has the remarkable consequence that *all* networks in the final population have the same phenotype (the same set of attractors), but slightly different genotypes. These small genotypic differences are reflected in the small, but not vanishing, standard deviation in the final distribution of sensitivities. In the next section we will come back to the structural differences that exist between the networks in the final population.

### Structure of the evolved networks

**Figure 6:**
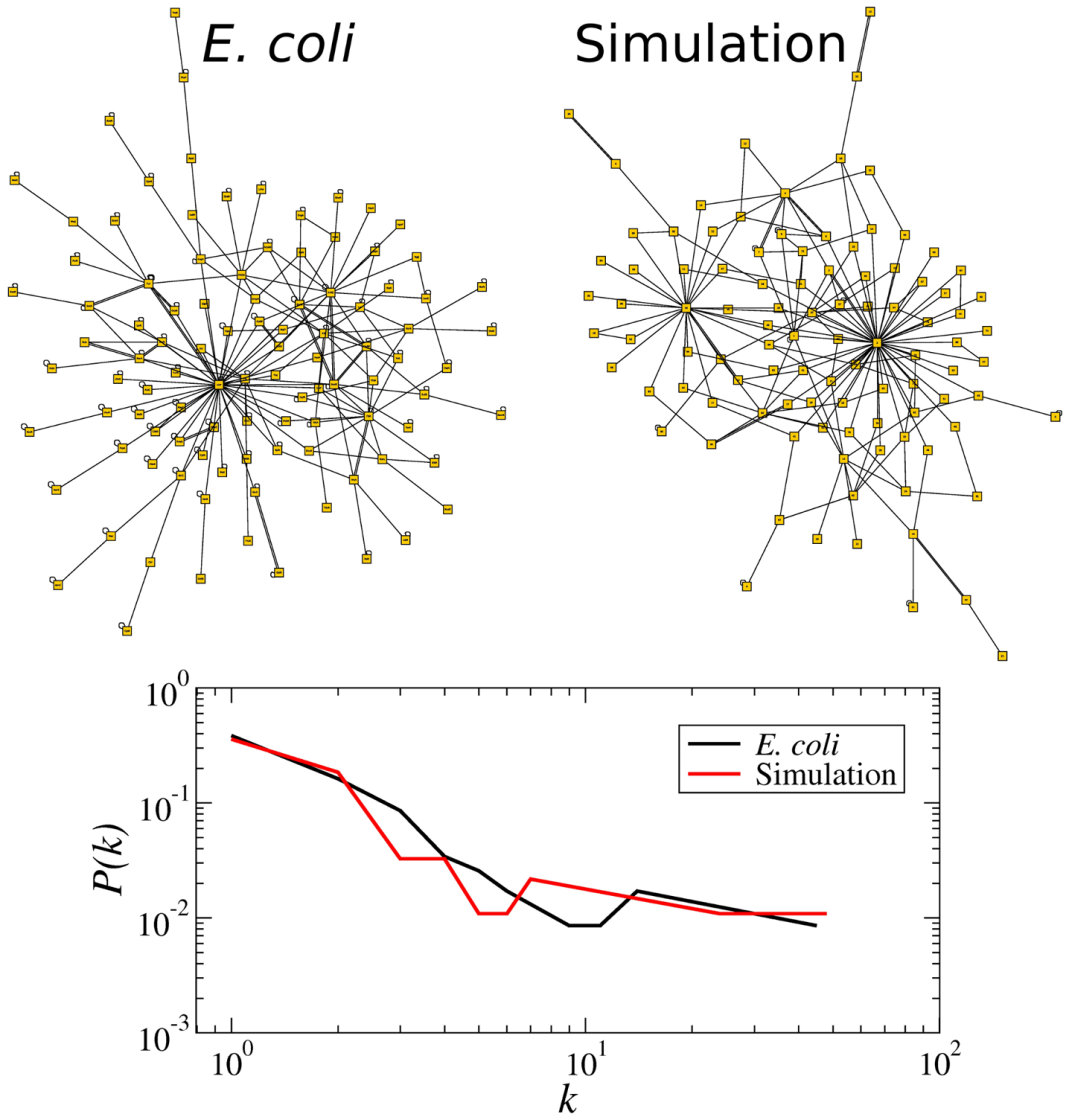
Network structure generated by the evolutionary process. The top-left network shows the structure of the giant component of the transcription factor interaction network of *E. coli* according to the RegulonDB [3]. This network has *N* = 101 nodes and average connectivity *K* = 2.46. The structure on the top-right corresponds to the typical network that results from our evolutionary algorithm, which in this particular case has *N* = 100 nodes and average connectivity *K* = 1.85. Note the existence of global regulators, i.e. nodes with a great number of output connections. The bottom panel presents in a log-log plot the out-degree distribution of these two networks to illustrate their remarkable similarity.

Of great interest is the structure (or topology) of the networks that survive until the end of the evolutionary processes, for such structure should encode the evolutionary trade-off that these networks were optimized for. We started the simulation with homogeneous random networks for which all nodes had the same number of inputs (in-degree) 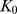 and where the number of outputs (out-degree) was Poisson distributed. However, at the end of the simulation the evolved networks contain *global regulators*, namely, nodes with a large number of output connections (targets), as illustrated in Fig. 6. In fact, the typical network structure produced by our evolutionary process was qualitatively similar to the structure of the giant component of the *E. coli* transcription factor interaction network [2], [3], [46] (Fig. 6). This structure is characterized by short-tailed in-degree distributions (Poisson or exponential) and long-tailed out-degree distributions. Such an outcome was unexpected for two reasons. First, the specific structure of the network was never explicitly considered in the selection mechanism nor did we implement any explicit re-wiring rule as in other models of network evolution [36], [54], [55]. Second and more importantly, global regulators introduce strong correlations in the network dynamics and it is not obvious that these correlations offer an advantage in surviving the selection pressure imposed by the ACC and AIC. Although the final networks are too small to accurately determine the out-degree distribution resulting from this evolutionary process (*N* = 100), the systematic occurrence of nodes with a high number of output connections (hubs) suggests that this type of network structure could also be an emergent property intimately related to the critical dynamics and evolvability of the network, as it has been suggested for other types of networks [56], [57]. It is important to mention that the existence of hubs in the evolved networks is not simply a consequence of the mutagenic algorithm because control networks that “evolved” without selection but subjected to the same type of mutations do not exhibit this characteristic (see Fig. S3).

**Figure 7:**
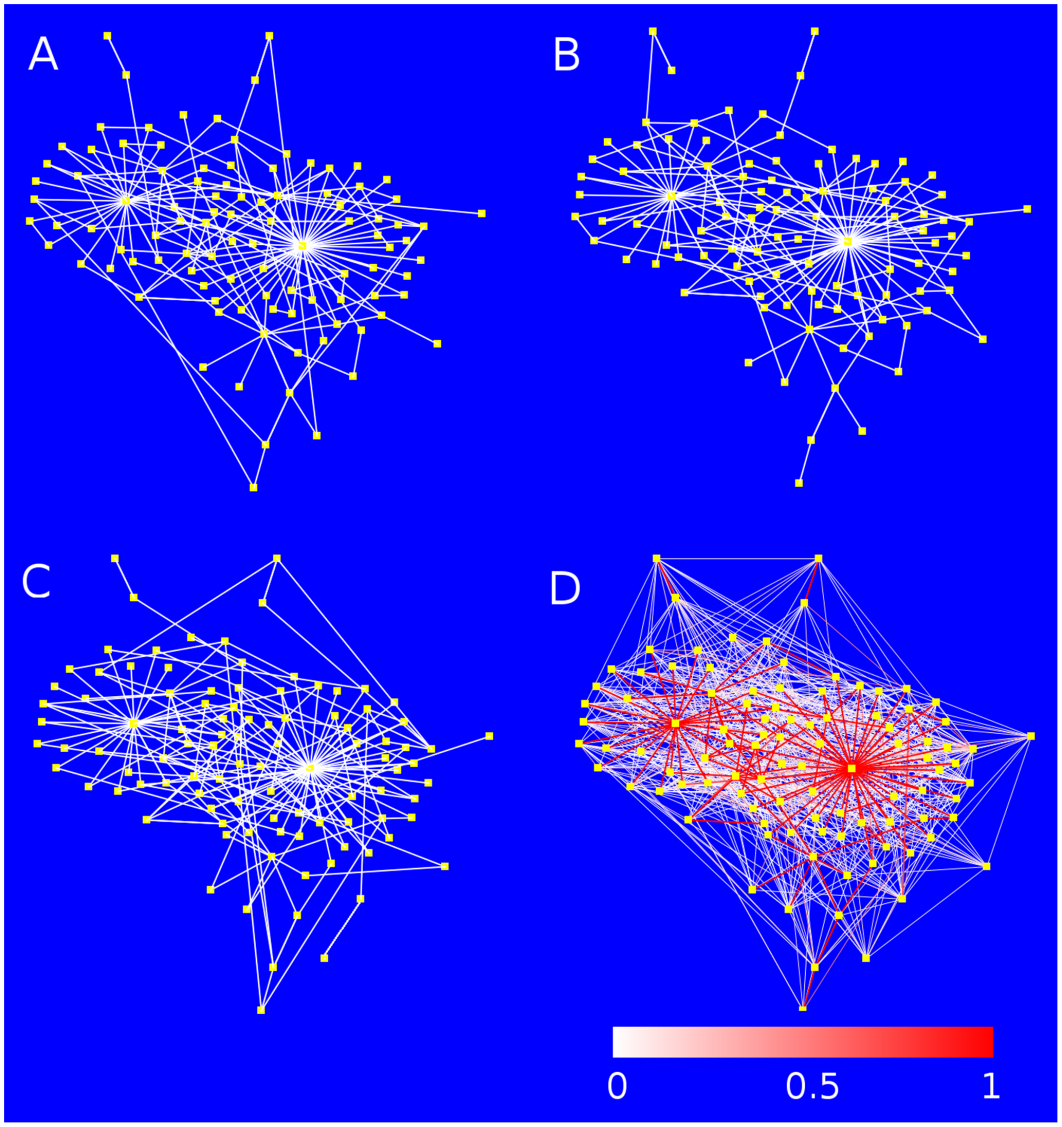
Structural variability within the population. Although all networks in the final population have the same attractor landscape, they are structurally not completely identical to each other. Here we show three networks randomly chosen from the final population (A, B and C). The image in D is a superposition of all the 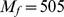 networks in that population. The *link persistence* is defined as 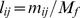, where 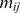 is the number of networks in the final population in which the nodes 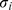 and 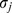 were connected. The links in D have been colored according to their persistence (white for 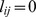 and red for 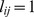). It is apparent that the highly persistent links mostly belong to the global regulators (hubs). This strongly suggests that the global regulators play an important role in determining the phenotypic landscape of the population.

As was mentioned before, although all networks in the final population had exactly the same attractor landscape, the networks themselves are not identical to one another. This is shown in Fig. 7, where three networks randomly chosen from the final population are displayed (A, B, and C). It is clear that, although similar, these networks are not identical. The final diagram D is a superposition of all the 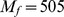 networks in the final population. Since all the final networks came from the same common ancestor, the genes in all these networks have the same evolutionary history. Therefore, it is possible to stack up these networks on top of each other and compare them. In order to measure the degree of similarity between these networks, we computed the fraction of occurrence 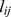 of the link between the nodes 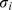 and 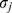 in the population, for all pairs *i* and *j*. Thus, if 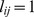 the two nodes 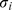 and 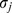 were connected in all the networks in the population, whereas if 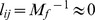 then these two nodes were linked only in one network of the population and disconnected in the rest of the networks. Very remarkably, Fig. 7D shows that the more persistent links in the networks throughout the population are the ones that belong to the global regulators.

**Figure 8:**
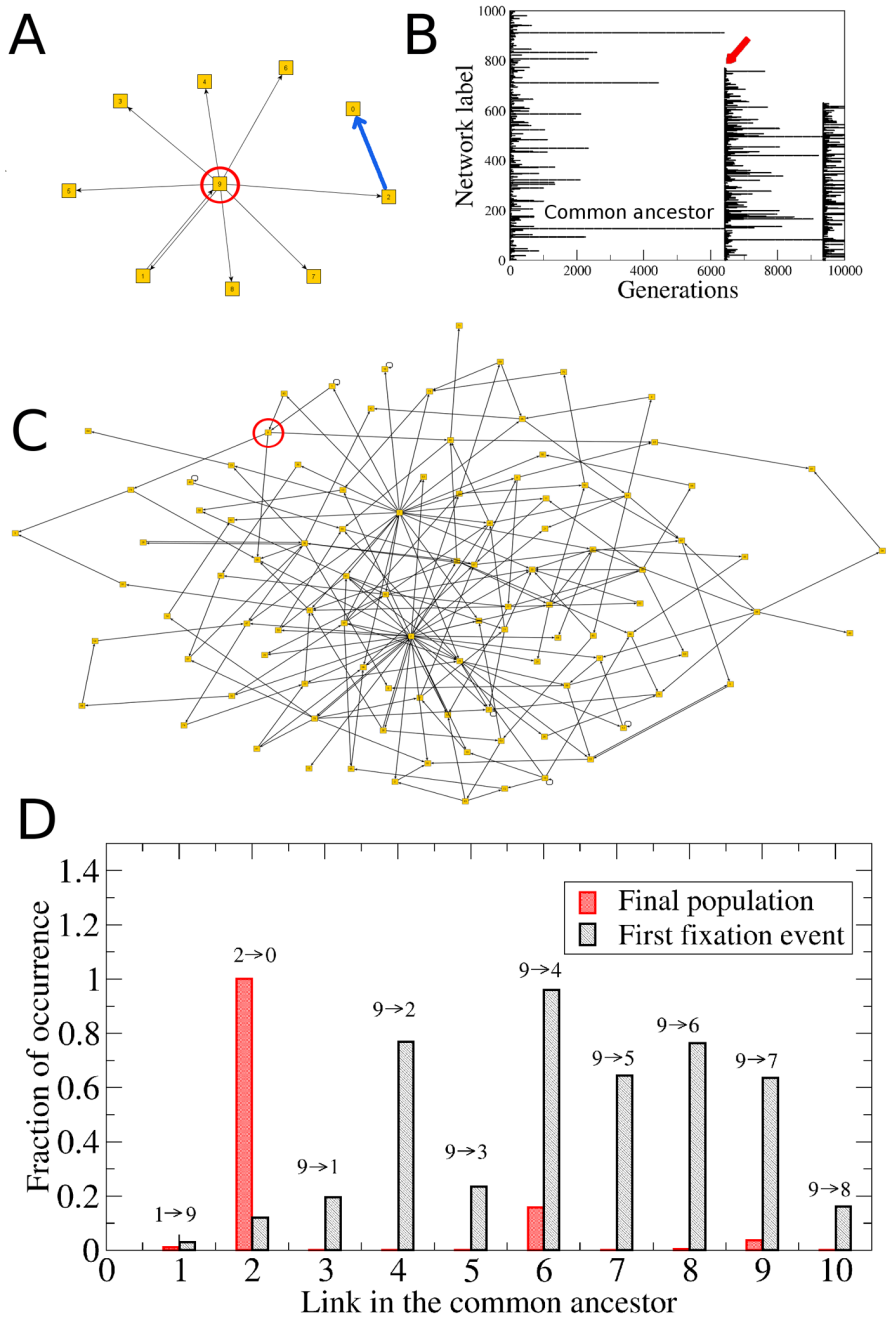
Evolution of the network topology. (A) The common ancestor network has 10 nodes and one of them (node number 9) is a global regulator that regulates 8 other nodes. (B) Diagram of strain survival times showing the first fixation event at generation *g* = 6411 (indicated by the red arrow). The common ancestor network is the one that gives rise to the population of the first fixation event. (C) Structure of a randomly chosen network in the final population (generation *g* = 250000). The initial hub (node number 9) is the one marked with the red circle. Note that at the end this is not a hub anymore, but just another ordinary node of the network. (D) Distribution of the link persistence 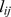 for the 10 connections 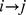 of the common ancestor. The black and red histograms represent the populations at the first fixation event and at the end of the simulation, respectively. Even after the first fixation event, the links 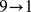, 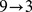 and 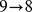 almost disappear from the population. Furthermore, in the final population none of the links of the initial hub occur at significant frequency. By contrast, link 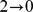 is present in all the networks of the final population because node 2 became a hub throughout the evolutionary processes. Link 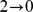 is indicated with the blue bold arrow in (A).

The existence of global regulators in the final networks raises the question as to whether the common ancestor network (from which all the other networks evolved) had, just by chance, some nodes with a “special” topological context that predestine them to eventually become the global regulators. For instance, it could be the case that the common ancestor network contained nodes with a number of output connections far above average. These initial hubs might have played an important role in controlling the network dynamics from the very beginning and therefore they may have remained being hubs throughout the evolutionary processes and end up as the global regulators observed in the final networks. To answer this question we performed simulations in which all the networks in the initial population were explicitly constructed with one node with a high number of output connections. Fig. 8A shows a typical example in which the common ancestor network has one hub that regulates 80% of the other nodes in the network (in this particular case the hub is node 9). However, at the end of the evolutionary process (generation *g* = 200000) this initial hub has turned into just another ordinary node in the network with no special characteristics (Fig. 8C). This can be seen more quantitatively in Fig. 8D, which shows, for each link 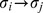 of the common ancestor network, the fraction of occurrence 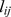 of that link in the entire population at two generation times: after the first fixation event (black histogram), and in the final population (red histogram). It is apparent from this figure that even after the first fixation event the initial hub has lost some of its connections in many networks of the population. At the end of the simulation processes none of its original connections significantly occurs in the final population. By contrast, two of the original nodes (nodes 2 and 7) without any special property become the global regulators in the final networks. Results similar to the ones reported in Fig. 8 systematically occurred in our numerical simulations, namely, the initial hubs in the common ancestor networks always lost their “hub” property throughout the evolutionary processes and ended up just as random ordinary elements in the final networks. Furthermore, very often the nodes that became the hubs in the final networks were not even present in the initial networks, but added later at some intermediate generation as a result of a duplication/divergence event.

### Importance of the α-fitness criterion

**Figure 9:**
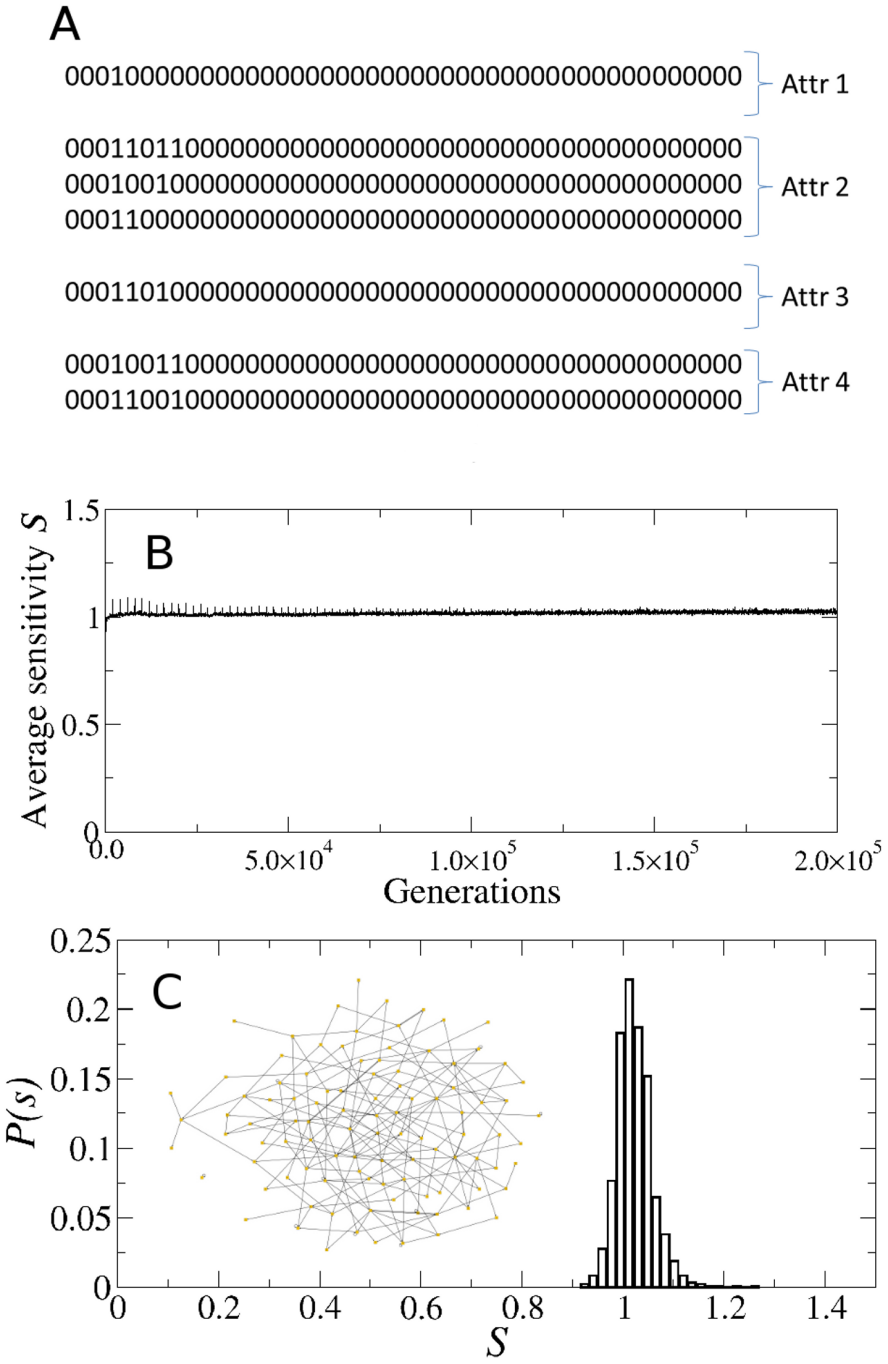
Importance of the gene expression variability as a fitness criterion. (A) Typical example of the attractors obtained when the evolution of the population is carried out without implementing the 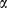-fitness criterion. In this particular case, instead of the 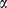-fitness we used the *S*-fitness which assigns a higher replication rate to the networks whose sensitivity is closer to 1. The final attractor landscape consisted of 91 attractors, and only 4 are partially shown here (only the first 45 digits in each attractor state are shown; the remaining 55 digits are all 0's). Note that only the first 10 digits in each attractor state show some activity. There are the 10 genes in the networks of the original population. (B) Plot of the average sensitivity of the population throughout generations, showing that the sensitivity very quickly approaches 1 and remains very close to 1. This is expected since we are explicitly selecting for networks with sensitivities 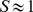. (C) Histogram of sensitivities in the final population (generation *g* = 200000), showing that most networks in the final population have become critical. The inset shows the structure of a typical network in the final population. Note that this network exhibits a more homogeneous random topology with no hubs. This is always the case when 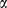-fitness is not used as selection criterion.

The *α*-fitness criterion was introduced to increase the reproduction rate and hence to favor those networks that exhibit high gene expression variability (information content) in their attractor states. If we perform the evolution of the networks solely by applying the ACC and the AIC but without using the *α*-fitness in the selection (which is equivalent to setting *α* = 1 for all networks), then all the surviving networks at each generation will generate the same number of descendants, equally contributing to the population at the next generation. Under such circumstances, the attractors in all the networks of the population will end with only zero values for σ, as shown in Fig. 9A (only the first 10 genes show some activity because they were the only ones present in the initial generation). This is mainly due to steps 1 and 3 of the mutation algorithm presented in the Methods Section which, together with the ACC, introduce a bias towards the state 0 in the Boolean functions. This in turn is needed to consider the physical meaning of the new Boolean functions: Each time a new gene 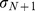 is added to the network (through a gene duplication), the extension of the Boolean functions of the target genes that have accepted the new gene 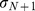 as their new regulator (input) is carried out by expanding the Boolean function's truth tables of each target gene as follows: Where in the configuration of the new expanded input vector (row in truth table) the new gene has value 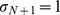 the output of that target gene is assigned 1 or 0 randomly; whereas when 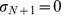 in the input vector, the output is kept equal as it was before the addition of the new gene because in that input configuration the new regulator is in the off state and does not contribute to the regulation. Consequently, it follows that a trivial way to fulfill the ACC and preserve the old attractors after the duplication event is by selecting networks in which the new gene is inactive (i. e. 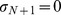) in all the attractors, since in this case the new part of the Boolean function is never used. Thus, without the *α*-fitness filter, all the new genes would appear in the 0 state in all the attractors. (This does not mean that in the transient states before the attractor is reached, the new genes cannot take, transiently, the value 1.)

However, it should be noted that even without the *α*-fitness we can still obtain critical networks as a result of the evolutionary process. To show that criticality does not depend on *α*-fitness we enforced the evolution of criticality and show that such networks do not exhibit *α*-fitness. Thus, we evolved populations of networks subjected to the ACC and the AIC as usual. But instead of using the gene expression variability *α* as the additional fitness parameter, we demanded sensitivity *S* to be close to 1 as a selection criterion. Specifically, we explicitly selected for criticality by making the replication rate of the networks proportional to 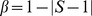. Thus, networks with *S*≈1 were replicated at a higher rate than networks with *S* far from 1 (networks with negative values of 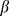 did not replicate). Fig. 9B shows the evolution of the average network sensitivity using this “*S*-fitness” criterion (together with the ACC and the AIC). As expected, the average sensitivity of the population very quickly approaches 1 and remains close to 1 throughout the evolutionary process. Fig. 9C shows the histogram of sensitivities in the final population (generation *g* = 200000). It is clear that this process generates critical networks with *S*≈1, although their attractor landscape (shown in Fig. 9A) has no information content whatsoever. Very remarkably, however, the networks produced in this way always exhibited random topologies with no hubs at all (see the inset in Fig. 9C). The networks developed hubs only when the *α*-fitness was used (together with the ACC and the AIC) and consequently the attractors exhibited genetic variability distributed around *α* = 0.5, as in Fig. 4A.

### Robustness of the evolved networks

**Figure 10:**
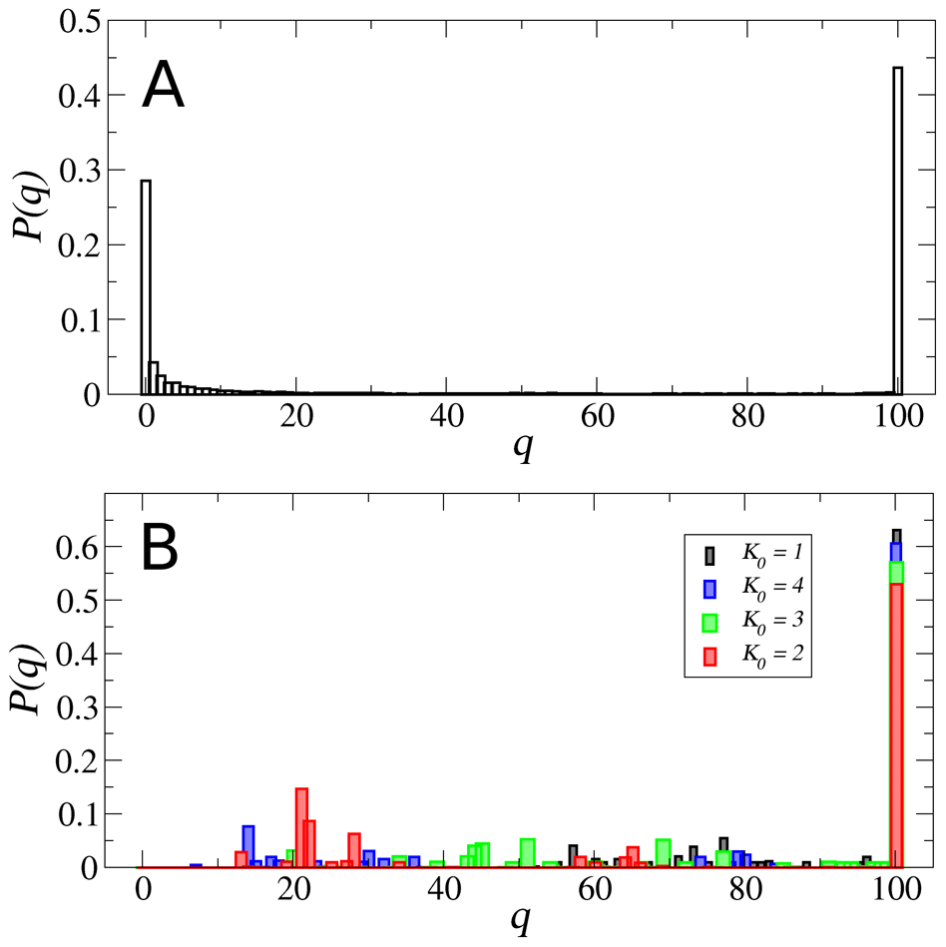
Robustness of the attractor landscape. Plots of the probability 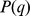 that a percentage *q* of the network attractors is conserved after a gene the knockout of one gene for critical networks constructed *de novo* (A) and the critical networks that result from the evolutionary process (B). The differently colored distributions in B correspond to populations that started in different dynamical regimes (as in Fig. 1). Note the high probability for the *de novo* networks to lose all their attractors by a gene knockout (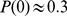) which does not happen for the evolved networks (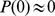 in all cases). Conversely the probability to conserve all attractors is considerably larger for the latter than for the former (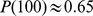 and, 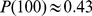 respectively). These data were computed from populations of 1000 networks and 500 attractors per network.

Since the evolved networks were selected to optimize the evolutionary trade-off, it is important to determine the robustness of their attractor landscapes under mutations. This robustness should be compared against the one observed in networks that are also critical, but that did not go through the evolutionary process. To measure such robustness, we removed one gene from the network and computed the probability 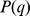 that a percentage *q* of the existing attractors is conserved as a result of this mutation. (We also implemented other types of mutations, such as rewiring or removing some input or output connections of one gene, or changing its Boolean function, and the results are qualitatively similar.) Each gene in the network and each network in the population was subjected to such a deletion mutation and analysis of its consequence. It should be mentioned that the networks in the final population had between 100 and 500 attractors (most likely the total number of attractors per network was higher but we worked with no more than 500 attractors per network—see the Methods section for a description about the search of new attractors). We also computed 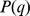 for critical networks of the same size (*N* = 100) as the evolved, but that were constructed *de novo* to be critical, namely, networks that were constructed with an initial sensitivity 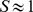 and did not undergo any selection process. Fig. 10 shows the probability 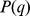 for the evolved networks (panel A) and the *de novo* networks (panel B). Note that following deletion of one gene, the *de novo* critical networks either conserve the entire attractor landscape (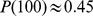), or none of the existing attractors is conserved (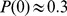). There are almost no other choices for these networks because 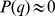 for intermediate values of *q* between 0 and 100. In contrast, the *evolved* critical networks do not exhibit such all-or-none behavior. Instead, with a very high probability all the existing attractors in the evolved networks are conserved (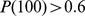), whereas for *q*<100 the probability 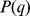, although small, was appreciably larger than zero. Remarkably, it never happened in our simulations that all the attractors of the evolved networks changed after the deletion of one gene, as it is apparent from Fig. 10A, i.e., 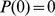. This last result indicates that, under the deletion of one gene, the evolved networks change only one fraction of their attractors but not all of them and that most likely, they will not change anything. The above behavior epitomizes mutational robustness and is consistent with knockout experiments in many organisms, which reveal that the knockout (or mutation) of one gene (node of the network) most of the time does not cause gross phenotype change.

## Discussion

Dynamical (phenotypic) robustness, the return to attractor states following perturbations of gene activities, and flexibility, the ability to switch between attractors, are two central properties common to all living organisms. While apparently opposed to each other, they jointly guarantee developmental robustness and homeostasis while allowing for developmental change and physiological adaptation to (i.e. intra-individual coping with) environmental fluctuations within the lifetime of an individual. Networks that are dynamically in the critical phase are poised between such phenotypic robustness and flexibility and have been shown to exhibit maximal information diversity to cope with changing environments [27], [34]. Here we show that the ontogenetically important coexistence of dynamical robustness and flexibility (the developmental trade-off) is related to an analogous balance between the opposing phenomena at the phylogenetic time scale: mutational robustness (preservation of attractor landscape following mutational network rewiring) and innovation (expansion of the attractor landscape). The selection for these two properties using the attractor conservation (ACC) and innovation (AIC) criteria as biologically plausible fitness filters in simulated network evolution led to networks whose structural properties and Boolean functions dictated a dynamically critical behavior (Fig. 3).

We should note that for the AIC in this evolutionary scheme, innovation of phenotypes occurs in two distinct ways. On the one hand, the generation of new attractors can be considered as the emergence of new phenotypes. On the other hand, the addition of new genes to the network also adds new information to the already existing attractors by modifying the attractor states and their basins. In either case, for this information to be useful and contribute to an organism's discriminatory response to variable environments, the new genes must have an activity that changes from one attractor to another. Therefore, a third *ad hoc* biologically motivated selection constraint we used was that the average variability of the genes in the attractor landscape must be significantly different from 0. Although this constraint is biologically important, it is not required for the evolution towards criticality because one can construct critical networks whose attractors have a nearly zero genetic variability (Fig. 9). However, only when the genetic variability of the attractor landscape was appreciably different from 0 did the networks evolve structures with global regulators (hubs). Indeed, based on our numerical simulations we can assert that the hubs emerge with high probability whenever the networks are forced to conserve attractors with high genetic variability, namely, with high information content. At this point this provocative statement is an empirical observation that we have not been able to fully quantify and deserves much more study. In any case, our results indicate that the emergence of hubs throughout the evolutionary processes is a consequence of the constraints imposed on the network dynamics and not of the structure of the common ancestor network. This is consistent with studies carried out for other types of networks in which the dynamical constraints strongly determine the network architecture [56], [57]. It is important to mention that we have not been able to identify any special structural property of the initial nodes of the common ancestor network that can predict which particular nodes will eventually become global regulators in the final networks. In fact, when we explicitly provided some of the initial nodes with a special property, such as with high output connectivity or a special type of Boolean function, that property was lost through the evolutionary process. We should also note that the mutation algorithm alone does not generate hubs either. For networks that undergo mutations and gene duplication events but without selection do not acquire this topological feature (see Fig. S3).

Another remarkable property of the global regulators was the high persistence of their links across the networks in the population. Although the attractor landscape was the same for all the networks in the final population (as they stem from the same common ancestor), there were structural differences between them (Fig. 7). These genotypic differences are reflected in the fact that some regulatory interactions (links) between pairs of genes are present in some networks but not in others. However, strikingly, the regulatory links invariably present in all networks of the population mostly belong to the global regulators, as Fig. 7D shows. This strongly suggests that these global regulators play a fundamental role in maintaining the phenotypic traits (attractors) across the population in spite of the small differences in network structure, and may be one of the reasons why this type of topology has been developed in real genetic networks. The importance of the hubs to maintain the phenotypic landscape across the population, as revealed in Fig. 7, is not a trivial result. For it has been shown that very often the hubs are not the key elements that influence dynamics of the network [58].

It is worthwhile calling into attention that in our simulations the attractor conservation and innovation criteria are not as stringent as one may think. The reason is that, due to computer limitations, the attractor landscape can be evaluated in full only for small networks of (e.g. 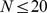). Thus, we completely determined the attractor landscape of all networks in the population only for the first generation. Thereafter, identification of new attractors was achieved by sampling a small fraction of the state space (at most 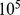 states for each network). Obviously we can apply the ACC and the AIC only to the attractors identified in such sampling and “hidden” attractors may exist that have been destroyed or created by mutation. In our simulations we worked with a maximum of 500 attractors per network. However, a more thorough search revealed that at the end of the evolutionary process, the evolved networks can have more than 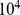 or even 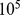 attractors (see Fig. S4). Thus, apparently we applied our selection criteria ACC and AIC to a small fraction of the attractor landscape, underestimating innovation and overestimating conservation. Quite remarkably, this was enough to generate criticality, robustness and hub-like structures.

The under-sampling of the state space in our numerical simulations has a biological equivalent because in reality selection does not act on all attractors which represent potentially realizable cellular phenotypes but rather, on the effectively existing phenotypes. For instance, for an organism like *E. coli*, with 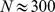 regulatory genes, it is very unlikely that all the 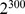 possible gene expression configurations have been explored by evolution – consistent with the notion that evolution is a quasi-non-ergodic process. Most likely, the search of new phenotypes (attractors) occurs by perturbing the already existing and occupied attractors. Thus the search is conducted in their state space neighborhood, which precisely reflects the algorithm we used to find new attractors (as described in detail in the Methods section). The idea that novel attractors must be reachable from existing ones that are already occupied by cells has wide-reaching consequence for the evolution of multi-cellularity and development [59].

Our approach does not study the evolution of evolvability *per se* but complements several studies of this question that use gene network-based computational models because we reverse the question: First, we do not impose an artificial “optimal“ phenotype (such as an arbitrary “equilibrium state” or attractor which networks are selected to maintain or evolve towards). Much to the contrary, the critical dynamics of our resulting networks was not an explicit selection criterion but is an independently known property of some networks that exhibit naturally high fitness. Second, we instead selected directly for properties related to evolvability, namely conservation and innovation of attractors, of which the former is of course directly related to phenotypic robustness. By not selecting for a particular phenotype through an artificially defined fitness value (as in [14]–[19]), we avoid exposing mutational robustness that simply reflects the known convergent mapping of many distinct network structures into one same phenotype (“equilibrium state”). Third, our selection criteria introduce the notion of global dynamics, embodied by the multi-attractor landscape as phenotype which, in contrast to the use of a single expression pattern as target phenotype, captures phenotypic adaptability and versatility of an organism. Quite interestingly, the critical networks produced by our evolutionary algorithm exhibit considerably higher mutational robustness than critical networks constructed *de novo*. Indeed, there is a high probability for the latter to change all their attractors after a simple mutation, whereas for the former this never happens (Fig. 4).

In conclusion, we do not present here a “molecular” mechanism, based on particular topological structures or mutations, to generate critical networks with global regulators. Instead, we propose a “dynamical” mechanism based on the conservation, innovation and information content of the attractor landscape. Our results show that dynamical criticality, a central property for the functioning of a living organism, naturally emerges as a consequence of evolution that favors evolvability. In other words, such an evolutionary process is sufficient for and robust in producing dynamical criticality. In our model such criticality appears as a coextensive property of evolvability and is not a direct adaptive phenotype. Whether evolvability in terms of our criteria ACC and AIC is necessary remains open. Specifically, we cannot exclude that in another evolution scenario there could be an adaptive component in the evolution of criticality, i.e. natural selection may directly favor networks with 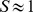. Then, the resulting networks could be associated with evolvability, in which case evolvability would be coextensive to a selected dynamical network property, i.e. a consequence of it rather than a direct result of selection, as proposed in other network models. The mutual relationship between dynamical criticality and evolvability remains thus to be evaluated carefully. But because evolution of multi-attractor dynamics and evolvability by network growth produces criticality, and since experimental evidence that existing gene networks are dynamically critical continues to accumulate, it is very likely that organisms that have evolved under the inevitable constraints of evolvability became critical.

## Methods

The following describes how the molecular nature of mutations, which affects gene regulation and its effector function, translates into a change in the Boolean network architecture. We describe the two classes of mutations at the two different time scales: (a) At the faster time scale, “point mutation” equivalents that alter the interaction properties of individual genes (nodes) through changes in either their regulatory or coding regions, which translate into altering the input or output properties of each node, respectively; (b) at the slower time scale the equivalent of larger scale genome rearrangements that is manifest as gene duplication event and corresponds to a duplication of a node that drives network growth.

### Point mutations

**Figure 11:**
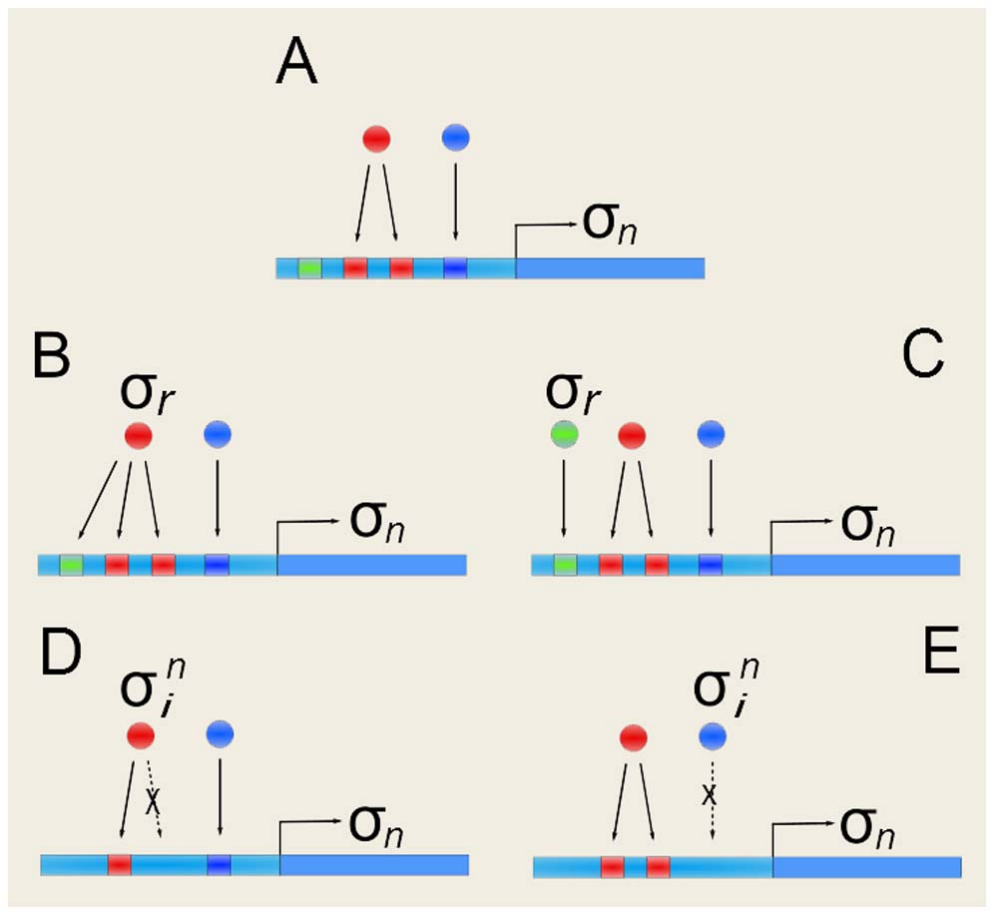
Mutations in the coding region of a gene. This type of mutation affects how the regulators of a given target gene 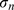 (inputs to node 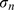) jointly control its output. (A) Gene 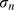 has acquired a new binding site, represented by the leftmost square in green. (B) The new binding site is occupied by one of the already existing regulators of 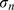. (C) The new binding site is occupied by a completely different gene (green circle) that thus becomes a new regulator of 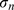. (D) Another mutation in the regulatory region is the deletion of existing binding sites. Here, the deleted site belongs to a regulator for which there are alternative binding sites left which thus remains a regulator. (E) Conversely, the removal of the binding site can completely break the regulatory interaction between 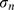 and the respective regulator. Deletion of binding sites that can leave a given gene with no regulators at all are not allowed.

To describe the mutagenic algorithm, in what follows we denote as 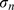 the gene that has been chosen for mutation and as 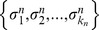 its set of 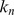 regulators whose activities {0,1} define the 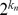 “input configurations” (or “entries”) to each of which the output value of 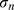 is assigned: 
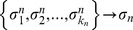
. Each gene in the network has two parts, a regulatory region and a coding region, as described and illustrated in Fig. 1. The regulatory region contains the binding sites for the regulators. Point mutations can be divided into two types, depending on whether they affect either the regulatory or the coding region of a gene, leading to distinct effects on the network and will be dealt with separately below. In the initial population, each gene has only one binding site (BS) for each of its upstream regulators. However, this situation changes through generations since new BS can be added, or some existing BS can be removed. The addition or removal of BS in the regulatory region is a mutation process that can be subdivided into the six types of mutation described below and illustrated in Fig. 11.

#### Mutations in the regulatory region

For a gene 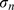 with 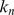 regulators there will be 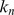 mutational events in its regulatory region. Each of this mutational events consists in one of the following alternatives: (i) 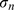 gains a new binding site in its regulatory region with probability 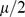; (ii) one of the already existing binding sites in the regulatory region of 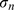 is randomly chosen and removed with probability 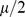; (iii) nothing happens in this mutational event with probability 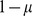. The addition and deletion of binding sites is carried out with restrictions as explained in what follows.

When a new BS is added to the regulatory region of 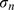, we have to decide how this new BS will be occupied (Fig. 11.A). For this, we randomly choose one gene from the entire network as the new upstream regulator of 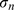. Let 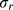 be this new regulator that will occupy the new BS in 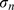. There are two distinct possibilities for 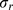:

1 The new regulator gene 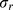 already belongs to the set of regulators 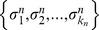 of 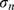 (Fig. 11.B). This occurs with probability 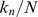. Let us assume that 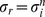 for some 
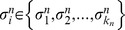
. In principle, when this happens the nature of the regulation 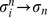 can change from activating to inhibitory, or vice versa. Alternatively, 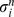 can become a dual regulator, being an activator or a repressor depending on which binding site it occupies. Therefore, this gain is implemented in our algorithm by randomly re-assigning the entries of the Boolean function 
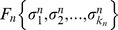
 only in those input configurations in which 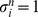. The entries of the Boolean function that correspond to 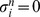 do not change.
2 Conversely, 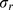 does not belong to the existing set of regulators of 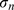 (which happens with probability 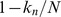). In this case, a new regulatory connection is created from 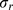 to 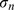 (Fig. 11.C). Consequently, the Boolean function 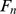 has to be extended to contain now 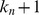 arguments: 
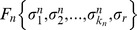
. This extension doubles the number of entries (input configurations) of the Boolean function and the new mapping of input configurations to output value is done in such a way that, in the entries where 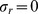, 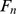 has the same values it had before the addition of the new BS, whereas the new entries for which 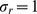 are assigned randomly. The addition of a new regulatory interaction that connects 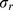 to 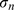 is accepted only if 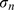 has less regulators than a given maximum, which in out simulations was set as 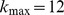. This yields a maximum of 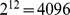 input configurations for each Boolean function. The reason for this maximum is to keep the algorithm within the limits of computer capacity, both in computing time and memory.

For the removal of a BS, let us assume that the selected BS to be removed is occupied by the regulator 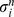. Then there are two possibilities:

3 There are additional BS for 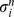 other than the one being removed (Fig. 11.D). Then, removal of one of these BS just changes the way in which 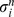 regulates 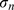. Therefore, in this case we randomly re-assign the entries of the Boolean function in which 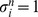. The entries for which 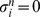 do not change.
4 There is only one BS left for 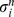. Therefore, removal of this BS eliminates the regulatory connection from 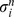 to 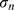 (Fig. 11.E). The number of regulators of 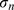 decreases by one and the number of entries of the Boolean function halves, retaining only the entries for which 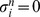. The entries where 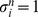 disappear from the Boolean function. To avoid generating genes with no regulators in the network, this mutation is accepted only if either 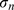 has regulators other than 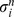, or if the number of BS for 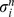 is larger than one. Thus, we do not allow nodes with no regulators in our algorithm.

#### Mutations in the coding region

**Figure 12:**
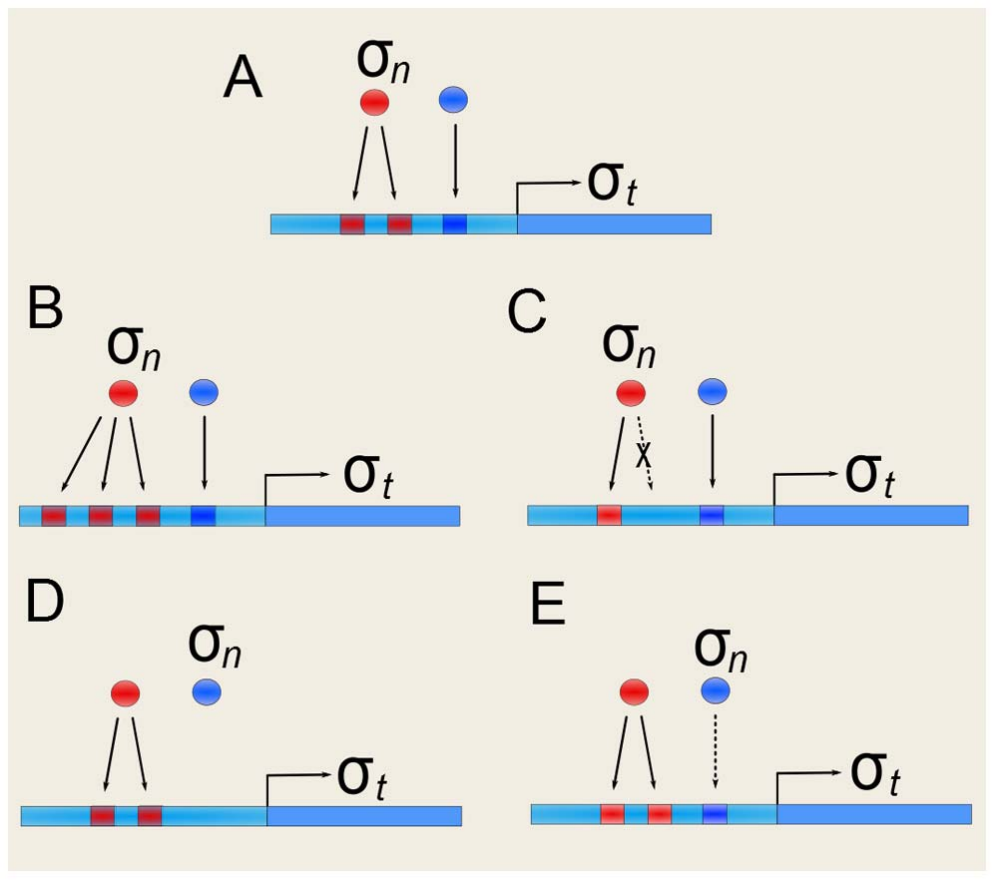
Mutations in the regulatory region of a gene. This second type of mutation affects the way in which a given gene 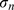 regulates its targets. (A) If 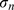 is one of the regulators of a target gene 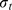. Then mutation in the coding region of 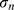 may afford gene 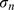 the capacity to bind to a new site in the regulatory region of the same target gene 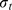 (B), or abrogate the capacity to bind to an existing binding site of that target gene (C). Conversely a change in the coding region of gene 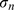 may provide new binding capacity for a site in the regulatory region of a new target, as it is shown in (D) and (E).

This type of mutation changes the way in which 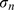 regulates its targets. Basically, this means that 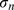 will gain or lose output connections, or modify some of the ones it already has. When a mutation occurs in the coding region of the regulator 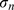 some other genes in the network will either lose or gain BS for 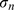. First we determined how many (potential) target genes will gain or lose BS for 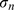 in a way that captures the fact that different regulators may have distinct affinities to their binding sites. To do this, let 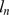 be the out-degree of 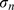 (i.e. its number of output connections). Then, we choose the number of target genes to be affected as 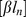, where 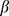 is a random variable uniformly distributed in the interval 0<β<1 and 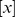 denotes the closest integer to 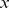. In other words, the number of target genes that will be affected by a mutation in the coding region of 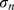 is proportional to its out-degree. This takes into account that highly promiscuous regulators, when mutated, will affect a larger number of genes than regulators that are less promiscuous and more specific. We performed simulations with different intervals for 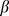, obtaining qualitatively similar results even for the narrower interval 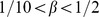. Once we have determined the number of target genes to be affected, with equal probability we decide whether all these 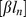 target genes will either gain or lose BS for 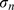 (one BS per target gene). Therefore, there are two cases:

5 Each of the 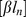 target genes gains a new BS for 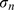. These target genes are chosen randomly from anywhere in the genome with uniform probability. Let 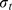 be one of these target genes. Then, we have to consider the two following possibilities (see Fig. 12):
  5.1

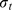
 belongs to the existing set of targets of 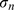 (which happens with probability 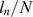). This means that 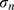 is already a regulator of 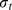. In such a case, we add a new BS to the regulatory region of 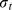 to be occupied by 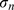 (Fig. 12.B), or we remove one of the BS in the regulatory region of 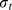 already occupied by 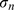 (Fig. 12.C). The addition or removal of BS in 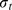 is done by following the rules described in mutations (1), (3) and (4).
  5.2

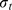
 does not belong to the set of targets of 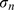 (this occurs with probability 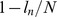). In this case we add a new BS to the regulatory region of 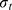 to be occupied by 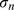 (Fig. 12.D and E). A new regulatory connection is thus established from 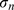 to 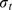, which is done as described in mutation (2).
6 Each of the 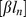 target genes loses an existing BS for 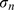. These target genes are then chosen randomly with uniform probability among the set of outputs of 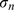. One BS is then removed from each of these outputs according to the rule given in points 3 and 4 above.

### Gene duplication and divergence

We implement gene duplication followed by divergence by randomly choosing one gene in the network and duplicating it. Let 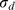 be the gene chosen for duplication. A duplication event increases the number of genes in the network from 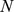 to 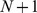, and 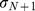 is the duplicated copy. The duplication can be performed in two different ways.

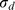
 duplicates with its own regulatory region. In this case, immediately after the duplication, the parent gene 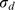 and its duplicate 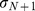 are identical in the sense that they first inherit the regulators, the Boolean function and targets from the parent gene. Then, we simulate genetic divergence by mutating the regulatory and coding regions of the duplicated gene 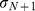 as described in the mutations (1)–(6) above.

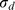
 duplicates without its regulatory region. Therefore, the duplicate copy 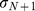 must be inserted into the transcription unit of another gene 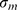. This means that the copy 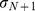 acquires the same regulators and Boolean function as 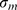, but having the coding region of the parent 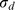 (the gene 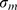 is not deleted or changed in any way). Then, divergence is simulated by mutating the coding region (and hence, altering the targets) of 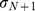 as described above. Note that when a new gene 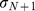 is added to the network, the targets of this new gene acquire a new regulator (which is the gene 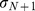 itself). Therefore, the Boolean functions of the targets have to be extended to incorporate the new regulator. This extension is carried out as described in the mutation step (2). As the network grows, it takes more and more computing time to find new attractors. Therefore, we grew the networks by gene duplication to a maximum of 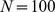 genes.

### Search of new attractors

In the simulations, the entire attractor landscape was known for all the networks in the initial population only. This is possible because the initial networks are relatively small (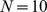) and the state space can be completely evaluated to find all the attractors. However, as the networks increase in size eventually only a random sample representing a small fraction of the state space can be evaluated. The search for new attractors was conducted only immediately after the gene duplication events, every 2000 generations. To find new attractors we dynamically explored the neighboring states of the attractors that we already had identified. This was achieved as follows: We set the network state to a state in one of its attractors. Then, we randomly perturbed 10% of the genes by bit-flips (change of activity values of the genes in that state) and evaluated the relaxation dynamics from that “perturbed” state for 60 time steps followed by evaluation of whether or not a new attractor was found. We performed this perturbation-based attractor search procedure 20 times for each network state in each of the attractors. When a maximum of 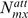 new attractors were found for a given network, the search was stopped for that network and the new attractors were incorporated in the expanded attractor landscape of that network. The attractor search was then continued with the next network in the population. For the results presented here we used 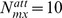, but similar results are obtained for 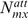 ranging from 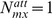 up to 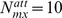.

The reason for stopping the attractor search (for a given network) when at most 10 new attractors were found was to keep the computing time within reasonable limits (the whole evolutionary process for a population of 1000 networks took on average 1.5 weeks). However, in addition to those attractors that we found and included in the attractor landscape of the networks, many more attractors were created after the duplication events. To obtain an idea of how many attractors were left out of the analysis of the evolving attractor landscapes, at the end of the simulation we performed a more exhaustive search by randomly sampling 10^6^ initial states in networks randomly chosen from the final population. Fig. S4 shows the number of attractors that were discovered in this search as a function of the number of sampled initial states. Surprisingly, in sampling 10^6^ initial states almost 150000 attractors were been found within only one network!! This number was much larger than the <200 attractors used to evolve these networks under the Attractor Conservation and Attractor Innovation criteria. It is remarkable that evaluating a small fraction of the attractor landscape (through the ACC and ACI) was sufficient to produce robust critical networks with global regulators. Note that this “agglomerative search” for new nearby attractor through single bit flip perturbations of existing attractors automatically detects “dynamically accessible” attractors – precisely as evolution of new phenotypes would have occurred that has to ensure that the latter are developmentally realizable [59].

## Supporting Information

Figure S1
**Reproducibility of the results.** Evolution towards criticality, as measured by the average sensitivity *S*, for 30 independent populations of networks. Initially, each population consisted of 1000 networks with exactly 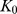 regulators per gene, where 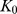 = 1, 2, 3, 4 (first four panels). Additionally, the fifth panel (labeled “varying connectivity”) shows six cases in which the initial networks had nodes with varying input connectivity, ranging from 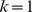 to 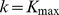, where 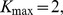
3, 4, 5, 6, 7. If for instance 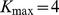 for a given network, this means that each node in that network could have 1, 2, 3 or 4 input connections with the same probability, yielding an average network connectivity 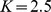. The last panel (bottom right) shows the histogram of sensibilities for the final networks in all these 30 simulations, which altogether encompass 18420 networks each with 100 nodes.(TIFF)

Figure S2
**Measures of criticality by means of Derrida maps.** A) Derrida maps of random Boolean networks operating in the ordered (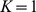), critical (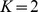) and chaotic (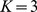, 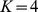) regimes. B) Derrida maps for 20 networks selected at random from the final population after 200000 generations of the evolutionary process (with mutation and selection). Note that all the curves are tangent to the identity close to the origin, which indicates that the final networks are critical. The data correspond to a simulation that started from a population consisting of ordered networks only (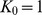). Panels C) and D) show similar results for initial populations consisting of critical (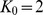) and chaotic (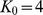) networks, respectively. In all the cases the Derrida maps clearly show critical behavior.(TIFF)

Figure S3
**Network structure after evolution.** Typical examples of the topology of the networks resulting from the evolutionary process after 200000 generations for two cases: (i) with mutation and selection (left column), and (ii) without selection (only mutations, right column). By “selection” we mean here the fulfillment of the ACC and ACI, and the implementation of the *α*-fitness. The labels 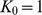, 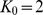 and 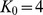 indicate the connectivity of the networks in the corresponding initial population. At the end of the evolutionary process, all the networks subjected to mutation and selection became critical and presented highly connected nodes (hubs). However, when only the mutagenic algorithm was implemented without selection, the final network structure was much more homogeneously random and no hubs were observed.(TIFF)

Figure S4 **Existence of hidden attractors.** At the end of the evolutionary process we randomly chose some networks of the final population and perform a “blind” search of attractors, which consisted in sampling 10^6^ randomly chosen initial states and determining the attractors each of these states lead to. This blind search revealed that the evolved networks had in fact much more attractors than the ones that participated in the evolutionary process. This figure shows a typical example of the number of “hidden” attractors found during the blind search as a function of the number of sampled initial states. In this particular case, the network had 95 “evolved attractors” (the ones subjected to the selection constraints ACC, AIC and *α*-fitness). However, note that after sampling 10^6^ initial states, almost 150000 attractors had been found and the curve does not seem to be flattening out. Thus, there are much more attractors in the evolved networks than the ones targeted by the Darwinian selection (the ACC, ACI and *α*-fitness) through the evolutionary processes.
(TIFF)

Text S1 **Measures of criticality by means of Derrida maps.** Derrida maps give information about the temporal evolution of the Hamming distance between two dynamical trajectories o the network. They represent another way to determine the dynamical regime where the network operates (in addition to the average network sensitivity *S*). Here we explain the concept of Derrida map and apply it to the networks that result from the evolutionary process. This analysis clearly shows that the evolved networks are critical.
(DOC)
