# Supplementary figures and images for: Criticality Is an Emergent Property of Genetic Networks that Exhibit Evolvability
Source: PLoS Comput Biol. 2012 Sep 6;8(9):e1002669. doi: 10.1371/journal.pcbi.1002669 (PMC3435273; doi:10.1371/journal.pcbi.1002669)

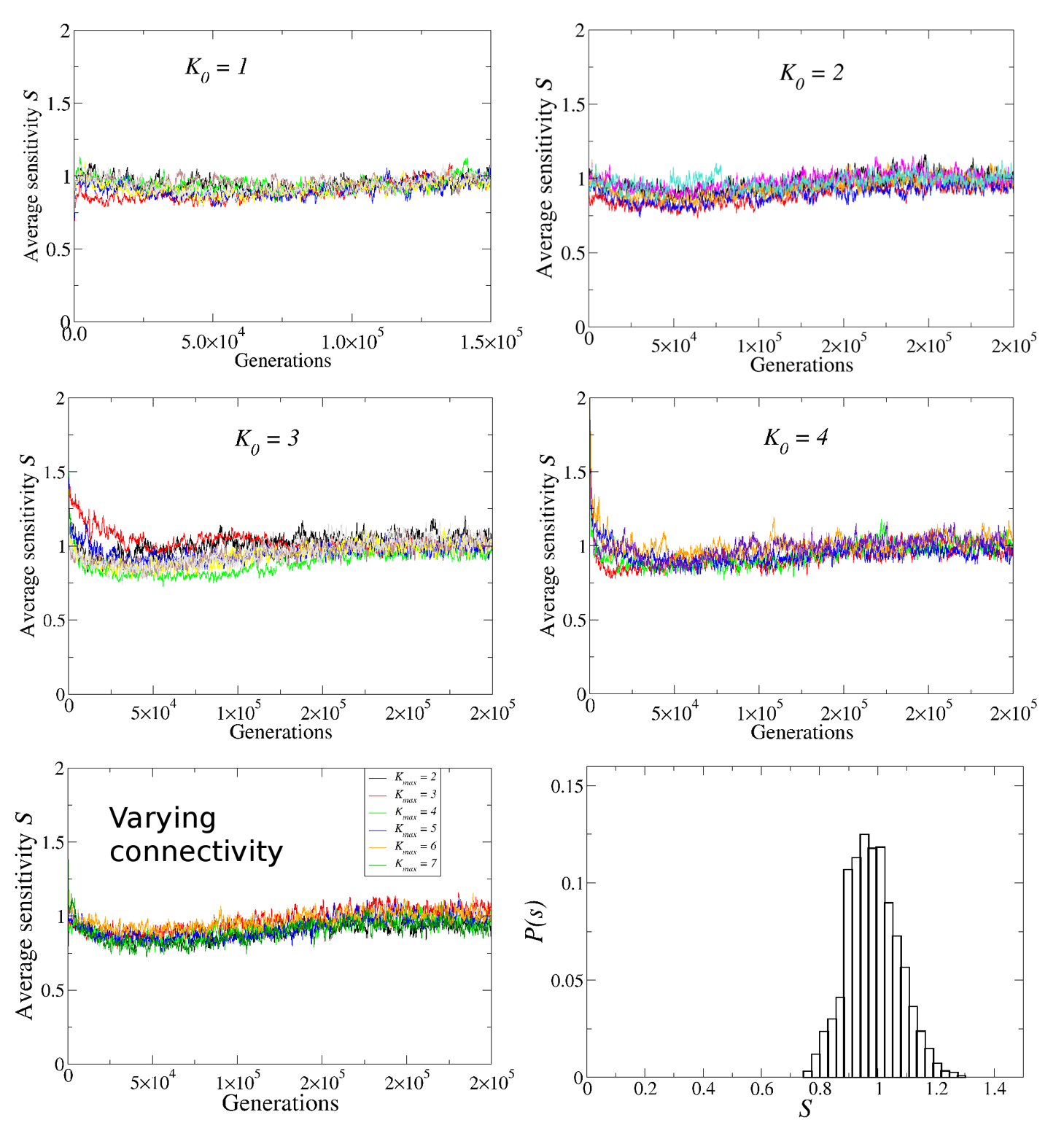

Supplement: Figure S1 — Reproducibility of the results. Evolution towards criticality, as measured by the average sensitivity S, for 30 independent populations of networks. Initially, each population consisted of 1000 networks with exactly regulators per gene, where = 1, 2, 3, 4 (first four panels). Additionally, the fifth panel (labeled “varying connectivity”) shows six cases in which the initial networks had nodes with varying input connectivity, ranging from to , where 3, 4, 5, 6, 7. If for instance for a given network, this means that each node in that network could have 1, 2, 3 or 4 input connections with the same probability, yielding an average network connectivity . The last panel (bottom right) shows the histogram of sensibilities for the final networks in all these 30 simulations, which altogether encompass 18420 networks each with 100 nodes. (TIFF) [file pcbi.1002669.s001.tiff]

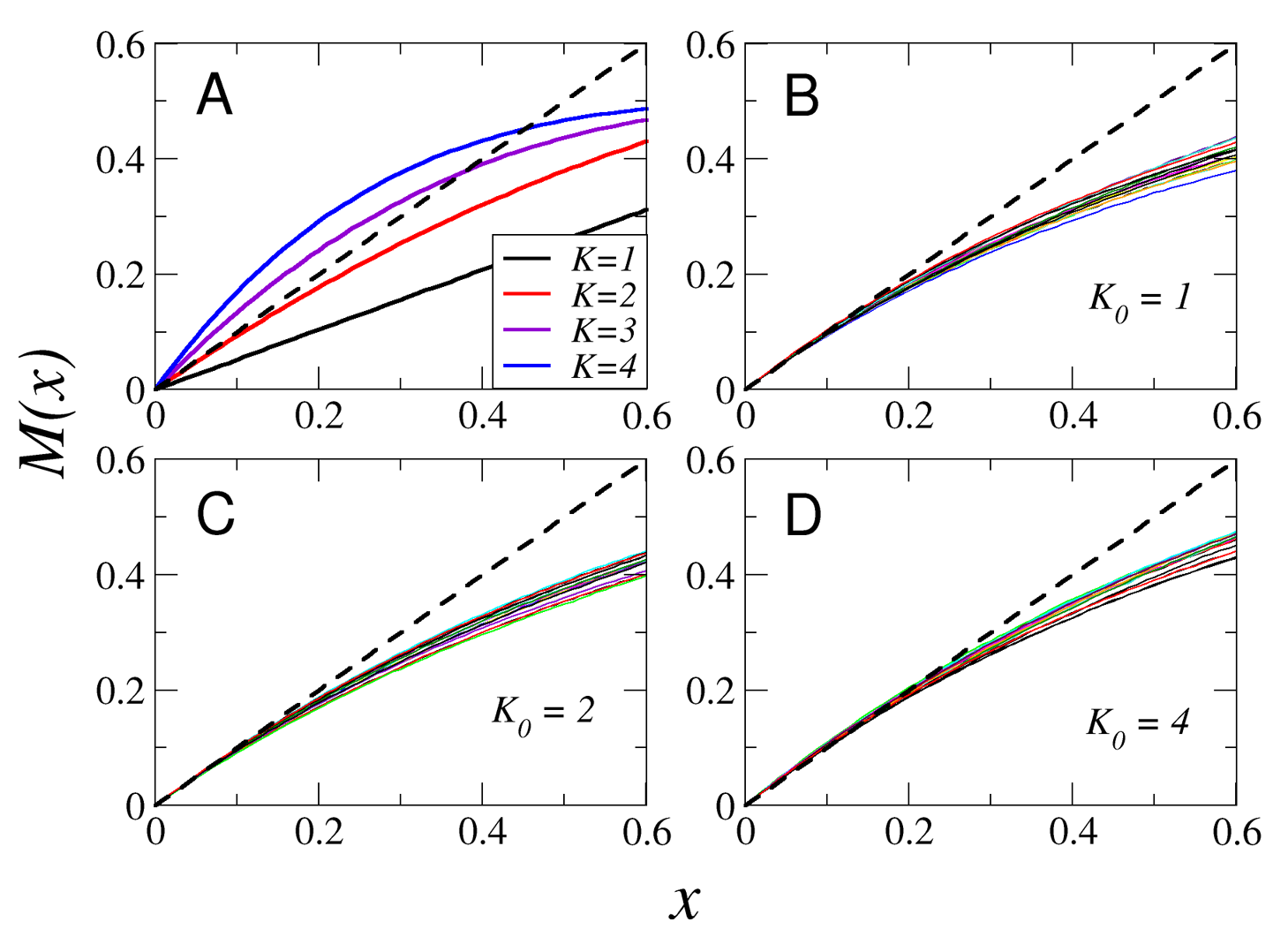

Supplement: Figure S2 — Measures of criticality by means of Derrida maps. A) Derrida maps of random Boolean networks operating in the ordered (), critical () and chaotic (, ) regimes. B) Derrida maps for 20 networks selected at random from the final population after 200000 generations of the evolutionary process (with mutation and selection). Note that all the curves are tangent to the identity close to the origin, which indicates that the final networks are critical. The data correspond to a simulation that started from a population consisting of ordered networks only (). Panels C) and D) show similar results for initial populations consisting of critical () and chaotic () networks, respectively. In all the cases the Derrida maps clearly show critical behavior. (TIFF) [file pcbi.1002669.s002.tiff]

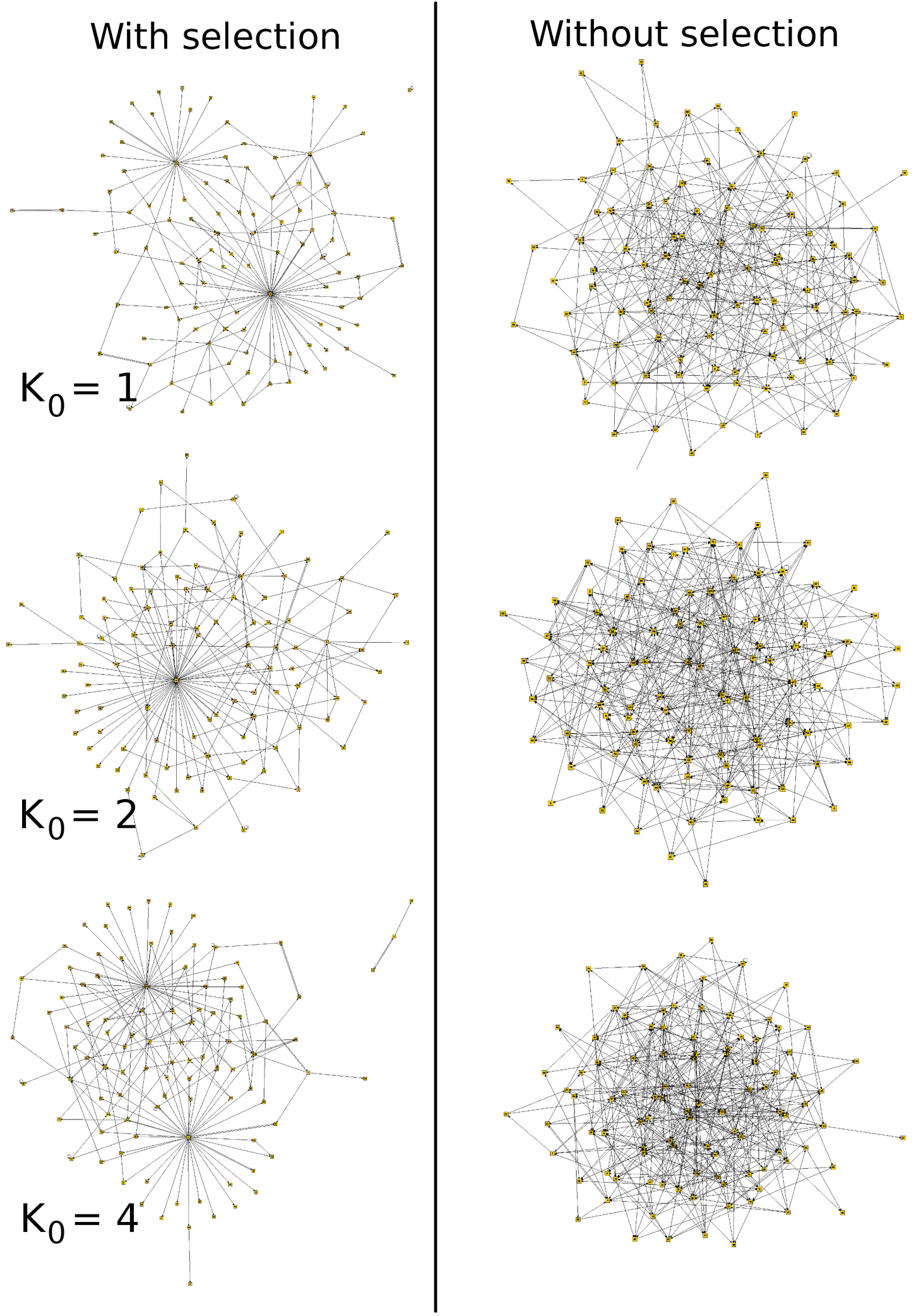

Supplement: Figure S3 — Network structure after evolution. Typical examples of the topology of the networks resulting from the evolutionary process after 200000 generations for two cases: (i) with mutation and selection (left column), and (ii) without selection (only mutations, right column). By “selection” we mean here the fulfillment of the ACC and ACI, and the implementation of the α-fitness. The labels , and indicate the connectivity of the networks in the corresponding initial population. At the end of the evolutionary process, all the networks subjected to mutation and selection became critical and presented highly connected nodes (hubs). However, when only the mutagenic algorithm was implemented without selection, the final network structure was much more homogeneously random and no hubs were observed. (TIFF) [file pcbi.1002669.s003.tiff]

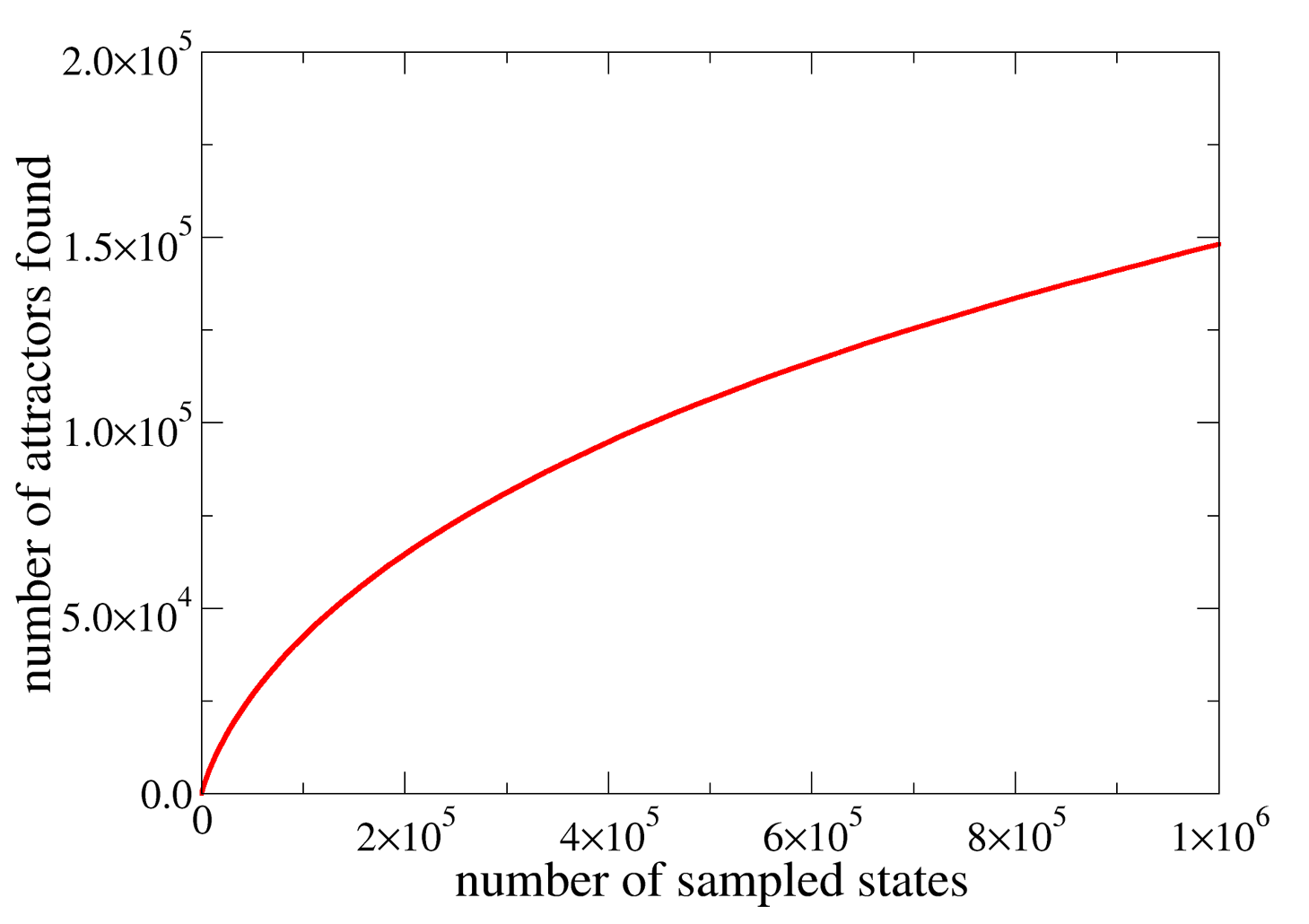

Supplement: Figure S4 — Existence of hidden attractors. At the end of the evolutionary process we randomly chose some networks of the final population and perform a “blind” search of attractors, which consisted in sampling 106 randomly chosen initial states and determining the attractors each of these states lead to. This blind search revealed that the evolved networks had in fact much more attractors than the ones that participated in the evolutionary process. This figure shows a typical example of the number of “hidden” attractors found during the blind search as a function of the number of sampled initial states. In this particular case, the network had 95 “evolved attractors” (the ones subjected to the selection constraints ACC, AIC and α-fitness). However, note that after sampling 106 initial states, almost 150000 attractors had been found and the curve does not seem to be flattening out. Thus, there are much more attractors in the evolved networks than the ones targeted by the Darwinian selection (the ACC, ACI and α-fitness) through the evolutionary processes. (TIFF) [file pcbi.1002669.s004.tiff]
