# Supplementary material for: Criticality Is an Emergent Property of Genetic Networks that Exhibit Evolvability
Source: PLoS Comput Biol. 2012 Sep 6;8(9):e1002669. doi: 10.1371/journal.pcbi.1002669 (PMC3435273; doi:10.1371/journal.pcbi.1002669)
Supplement: Text S1 — Measures of criticality by means of Derrida maps. Derrida maps give information about the temporal evolution of the Hamming distance between two dynamical trajectories o the network. They represent another way to determine the dynamical regime where the network operates (in addition to the average network sensitivity S). Here we explain the concept of Derrida map and apply it to the networks that result from the evolutionary process. This analysis clearly shows that the evolved networks are critical. (DOC) [file pcbi.1002669.s005.doc]

**Text S1. Measures of criticality by means of Derrida maps.**

The Derrida map characterizes the dynamical regime in which the network operates: ordered, critical or chaotic [1,2]. This map gives the temporal behavior of the Hamming distance between two distinct dynamical trajectories that started from different initial conditions. To define the Hamming distance, let us assume that we start the dynamics from the initial condition$\left\{\sigma_{1}(0),\sigma_{2}(0),\ldots,\sigma_{N}(0)\right\}$, which then generates the trajectory

$$\left\{\sigma_{1}(0),\ldots,\sigma_{N}(0)\right\}\to\left\{\sigma_{1}(1),\ldots,\sigma_{N}(1)\right\}\to\left\{\sigma_{1}(2),\ldots,\sigma_{N}(2)\right\}\to\cdots\to\left\{\sigma_{1}(t),\ldots,\sigma_{N}(t)\right\}\to\cdots$$

Analogously, the different initial condition generates the trajectory

The Hamming distance $x(t)$ is then defined as

.

It turns out that the temporal evolution of is determined by the dynamical mapping

,

where , the Derrida map, depends on the particular structure and Boolean functions of the network. It has been shown that the network sensitivity used in the main text as a measure of criticality is the slope of at :

.

Fig.S2.A illustrates the form of the Derrida map for random networks with nodes operating in the ordered (), critical () and chaotic (,) regimes. Note that only for critical networks becomes tangent to the identity line close to . On the other hand, Figs.S2.B shows the Derrida maps for 20 networks selected randomly from the final population that results from the evolutionary process with mutation and selection after 200000 generations. Note that these maps undoubtedly show that the networks in the final population are critical. The data shown in Fig.S3.B correspond to a simulation in which the initial population consisted of networks with initial connectivity . Analogous results are shown in Fig.S2.C and D for initial populations with networks in the critical () and chaotic () regimes. Regardless of the dynamical regime in which the networks of the initial populations operate, the evolutionary process developed in this work produces populations of critical networks.

**References.**

1. Aldana, M. Dynamics of Boolean networks with scale-free topology. *Physica A* **185**, 45-66 (2003)
2. Derrida, B. & Pomeau, Y. Random Networks of Automata - A Simple Annealed Approximation. *Europhys. Lett.* **1**, 45–49 (1986).
